# Axis I Psychiatric Disorders and Substance Abuse: A Systematic Review of Neuroimaging Findings

**DOI:** 10.3390/jcm14072156

**Published:** 2025-03-21

**Authors:** Bernardo Sosa-Moscoso, Alina Rivadeneira-Limongi, Filip Moncayo, Enrique Loor-Vera, Diana Álvarez, Lucia Geannett Vasquez Mena, Jose A. Rodas, Jose E. Leon-Rojas

**Affiliations:** 1 NeurALL Research Group, Quito 170157, Ecuador; bsosa413@gmail.com (B.S.-M.); alinarivadeneira61@gmail.com (A.R.-L.); filipmoncayowa@gmail.com (F.M.); enriquegloor@gmail.com (E.L.-V.); 2 Cerebro, Emoción y Conducta (CEC) Research Group, Escuela de Medicina, Universidad de las Américas (UDLA), Quito 170125, Ecuador; dianaluceli.alvarez@udla.edu.ec; 3 Facultad de Humanidades y Ciencias de la Educación, Departamento de Pedagogía, Universidad de Jaen, 23071 Jaen, Spain; lgvm0001@red.ujaen.es; 4 School of Psychology, University College Dublin, D04 V1W8 Dublin, Ireland; josea.rodasp@gmail.com; 5 Escuela de Psicología, Universidad Espíritu Santo, Samborondón 092301, Ecuador

**Keywords:** substance abuse, axis I disorders, neuroimaging, structural, psychiatric

## Abstract

**Background/Objectives**: The present systematic review analyses the neuroradiological findings in subjects with axis I psychiatric disorders (i.e., bipolar, major depressive, schizophrenic, anxiety, and post-traumatic stress disorders) and comorbid substance use disorder in order to elucidate the organic changes that occur in the brains of people suffering from both conditions. **Methods**: We analysed and compared the different neuroimaging findings extracted from 93 studies and 10,823 patients; articles were obtained from three databases (Scopus, PubMed [Medline], and the Cochrane Controlled Register of Trials [Central]) and subjected to specific eligibility criteria. We selected articles that assessed patients with axis I psychiatric conditions and a comorbid substance abuse disorder; articles had to report relevant neuroimaging findings and bias was assessed via the Newcastle–Ottawa scale. **Results**: Significant findings were found on the structure or function of psychiatric patients’ brains with comorbid substance abuse, with certain key areas that were further affected by substance use, especially in areas involved in reward processing, with reductions in volume and connectivity and the augmentation of stimuli-related activity. **Conclusions**: These results present important implications on the current understanding of psychiatric disorders and comorbid substance use, on the importance of neuroradiological tools in the diagnosis and treatment of these disorders, and on the search for potential new targets for the treatment of psychiatric disease and substance addiction.

## 1. Introduction

In our modern society, mental health has become an issue of ever-increasing proportion. Heightened awareness of psychosocial health and other psychopathological illnesses has led to an increase in the diagnosis of a wide range of psychiatric problems. In fact, research on the prevalence of psychiatric disorders in Africa has demonstrated a lifetime prevalence in mood disorders of up to 9.8% and up to 13.3% when looking at substance use disorders [1]. Moreover, mood disorders frequently co-occur with substance use disorders [2]; additionally, it has been demonstrated that the challenges in managing individuals with substance use disorders and other psychiatric diseases are analogous, if not identical [3].

These two disorders can co-occur more often than previously thought; studies have reported varied degrees of prevalence, with some reporting that 47% of people with schizophrenia have a serious substance abuse problem with drugs such as cannabis, tobacco, cocaine, or alcohol compared to 16% of the general population [4]. Moreover, substance use disorder and psychiatric problems significantly affect patients’ quality of life and, on a broader scale, exert considerable economic and societal repercussions [5]. The presence of comorbid substance use disorders and other psychiatric diseases exacerbates the patient’s symptoms, diminishes positive treatment outcomes, and heightens the chance of relapses, hence amplifying the impact of these comorbid conditions on the patient’s quality of life [2].

Given the impact of mental health conditions (including substance use disorders), their early and accurate diagnosis becomes paramount to ameliorate their deleterious effects on quality of life. However, mental disorders have always been diagnosed in a more traditional, clinical fashion, without the support of other diagnostic tests [6]. The Diagnostic and Statistical Manual (DSM) has been the epitome of this method, currently being the most used guideline to diagnose psychological and psychiatric disorders [6]. While the need for more accurate ways to diagnose psychiatric disorders is becoming increasingly evident [7], and attempts to compile research results have been ongoing for at least 35 years to date [8], there still exists a deficit of objective diagnostic criteria and guidelines that effectively combine clinical and radiological criteria to show the organic changes in psychiatric conditions (i.e., structural or connectivity changes in the nervous system) [9,10].

Taking these precedents into account, in this article, we comprehensively reviewed the findings of neuroradiological (namely, magnetic resonance imaging [MRI]) studies in patients afflicted with psychiatric disorders with comorbid substance use. Thus, we will compile the different alterations in brain morphology, circuitry activation, white matter alteration, and neurometabolite disturbances in these patients in hopes of finding a tangible difference or similarity between mental disorders and, in time, to lay the groundwork for the potential use of neuroimaging as a biomarker in psychiatric diagnostics.

## 2. Materials and Methods

The protocol of our review was registered in PROSPERO, with ID number CRD42021253070, and we followed the 2020 PRISMA guidelines.

### 2.1. Eligibility Criteria

We included case reports, case series, cohort, and cross-sectional studies, which were considered if they included patients with psychiatric axis I disorders (anxiety, mood, bipolar, psychotic, and eating disorders) that were assessed with neuroimaging—specifically with MRI (i.e., structural MRI, functional MRI [fMRI], MRI-spectroscopy, diffusion-weighted MRI [DW-MRI], Tractography). We excluded reviews or studies that considered patients with co-morbid neurological or non-axis I psychiatric disorders; studies with a high risk of bias or important methodological flaws were also excluded.

### 2.2. Information Sources and Search Strategy

All studies from inception until 2 April 2021 recovered from three databases (PubMed [Medline], Scopus, and the Cochrane Controlled Register of Trials [CENTRAL]) and written in English, Spanish, or French, were considered; grey literature databases were not included in this search. The key terms used in the search formulas included neuroimaging, magnetic resonance imaging, feeding disorders, mood disorders, anxiety disorders, bipolar disorders, psychotic disorders, axis I disorders, and substance use disorders combined with Boolean operators and medical subheadings (MeSH). The complete search strategy, for each database, can be found in the Appendix.

### 2.3. Selection Process

Two independent and blinded authors with knowledge of psychiatric disorders and neuroimaging assessed all the titles and abstracts against our eligibility criteria; any discrepancies were resolved by a third author and by mutual consensus. Finally, a full-text review of the filtered articles was performed in a similar fashion and the reasons for exclusion were recorded.

### 2.4. Data Collection Process and Data Items

Data were extracted manually from all the articles that successfully passed our selection process by the same authors that had filtered them originally; the data to be collected and extracted were discussed between all the authors and included the author, year, DOI, study design, demographic characteristics, psychiatric disease characteristics, neuroimaging characteristics, type of MRI technique used, and substance use disorder characteristics. The quality of the data extraction was verified by discussion during round tables between all authors.

### 2.5. Bias Assessment

We assessed the risk of bias in each study by applying the Newcastle–Ottawa Scale (NOS), which stratifies the risk into low, medium, and high [11]; if articles presented a high risk of bias, they were excluded. To ensure the quality of this assessment, the bias of the articles was independently screened by two reviewers; disagreements were resolved by a third independent reviewer.

### 2.6. Effect Measures and Synthesis Methods

Our study aimed to showcase the characteristics of neuroimaging findings in patients with axis I disorders and substance abuse through the means of a qualitative systematic review reporting absolute and relative frequencies as our outcome measures; therefore, a meta-analysis was not feasible due to the significant heterogeneity that occurred due to the inclusion of different pathologies and imaging modalities. Our included studies were tabulated based on the type of axis I disorder into schizophrenia, bipolar, depression, anxiety, and post-traumatic disorders; these groups were further subdivided according to the type of substance being used (i.e., cannabis, alcohol, opiates, etc.) and the neuroimaging modality (i.e., structural MRI, functional MRI, diffusion-weighted MRI, etc.).

## 3. Results

### 3.1. Study Selection

**Figure 1:**
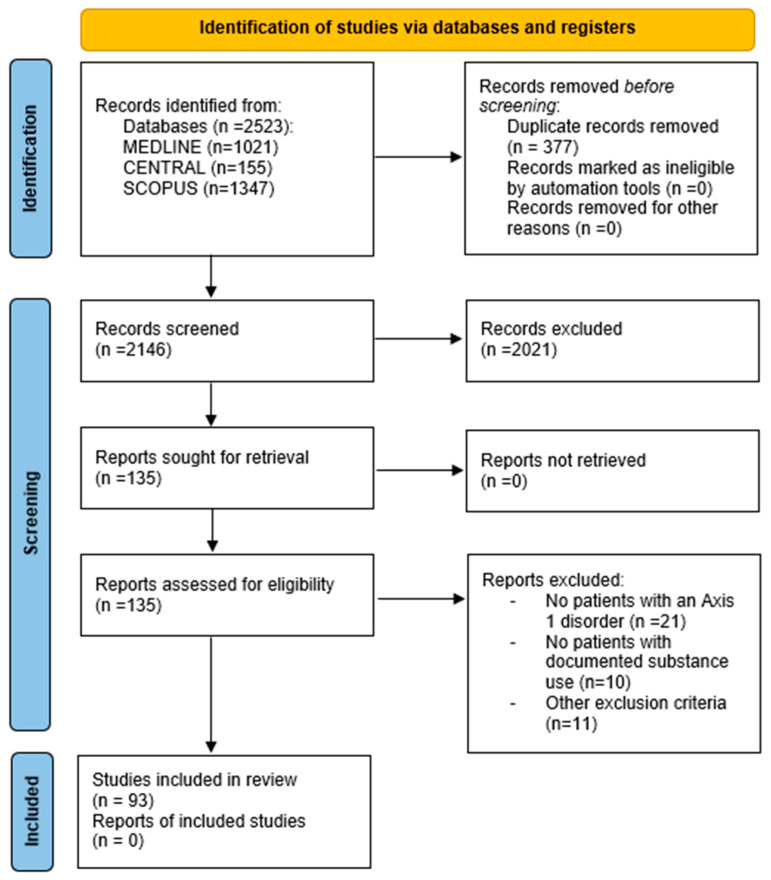
PRISMA flowchart showcasing the article selection process.

Our search strategy yielded a total of 2146 articles after duplicate removal; once the selection process was completed, 93 articles were deemed eligible for our review [12,13,14,15,16,17,18,19,20,21,22,23,24,25,26,27,28,29,30,31,32,33,34,35,36,37,38,39,40,41,42,43,44,45,46,47,48,49,50,51,52,53,54,55,56,57,58,59,60,61,62,63,64,65,66,67,68,69,70,71,72,73,74,75,76,77,78,79,80,81,82,83,84,85,86,87,88,89,90,91,92,93,94,95,96,97,98,99,100,101,102,103,104]. The complete selection process can be seen in the PRISMA flowchart (Figure 1).

### 3.2. Risk of Bias

The risk of bias assessment, with the NOS grading system, can be found in Table 1; a maximum of nine stars can be awarded in total (a maximum of four stars for selection, two for comparability, and three for exposure/outcome). In the NOS scale, a score of 9 represents the lowest risk of bias and a study is considered to have a high risk of bias if 3 or fewer points are assigned to it. In our review, there were no studies with this score, the lowest being a score of 5 assigned to 6 studies out of 93. For this reason, no studies were excluded due to a high risk of bias. Additionally, it is important to note that there were no differences in risk of bias between articles that dealt with different MR study types or different psychiatric disorders, which could have affected the conclusions we present in this review.

### 3.3. Schizophrenia and Substance Use Disorder

#### 3.3.1. Cannabis and Volumetric/Morphometric MRI

Most of the articles that studied the effects of schizophrenia (SZ) and other psychotic disorders with cannabis use or abuse determined a reduction in the size of the cerebral cortex and in total white and grey matter volumes. Some articles found a significant reduction in overall grey matter density (GMD) and volume in patients diagnosed with SZ and psychosis who had used cannabis [12,13,14]. Other studies found that the cerebellum showed a GMD decrease in schizophrenic cannabis users (specifically located in the right lobules III, IV, and V) and that this was related to the age of onset [15]. However, when comparing different groups over a 5-year period, a greater grey matter loss with ventricular enlargement was found in SZ patients with concomitant cannabis use against those without cannabis use [21]. Furthermore, when looking at cortical thickness, the study by Hartberg et al. (2018) revealed that cannabis users had reduced cortical thickness, especially in the middle frontal, right fusiform, and left superior gyrus [26], whereas Rais et al. (2010) found reduced cortical thickness in all SZ patients who had concomitant cannabis use in the right supplementary motor, inferior frontal, occipital, superior temporal, angular, and parietal cortices, as well in the whole cortical volume [27]. However, on the other hand, a study of fifty young individuals (23.9–mean age) diagnosed with a first psychotic episode reported no change in grey matter volume or white matter microstructure between cannabis users and non-users; this lack of association was maintained even when looking at heavy cannabis users [48].

However, when analysing specific regions of interest (ROIs) related to SZ, the study by Quinn et al. (2018) determined that patients had lower grey matter volumes in the bilateral precentral gyrus, right medial frontal cortex, right visual cortex, right occipital pole, right thalamus, bilateral amygdala, and bilateral cerebellum, regardless of cannabis use [17]. In contrast, a different study found a significant association between cannabis use and a decrease in cingulate cortex (CC) volumes (i.e., the bilateral posterior CC and left anterior CC) in patients with a first psychotic episode and in those with an at-risk mental state [18]. In a similar fashion, Solowij et al. found a reduction in volume in both cerebellar and hippocampal white matter, which was related to the cumulative dose of cannabis and the duration of SZ in the studied subjects [19,20]. Other studies have also found a reduction in grey matter volume in cannabis users with SZ in the left prefrontal cortex, left frontal gyrus, bilateral hippocampus and parahippocampal region, and anterior cingulate cortex [33,34]; an effect of such structural changes on executive function performance has also been reported [36]. Some studies have postulated that the effect on cortical volume is due to cannabis alone and that it is independent of the psychiatric condition [35], while other authors have described a different effect of cannabis use in these groups, reporting an increase in the volume of the lenticular nuclei [37] and of the left parietal, orbitofrontal, and frontal lobes, as well as of the bilateral hippocampi in SZ patients [32].

Certainly, one of the most studied ROIs (in terms of volumetry) in SZ patients with concomitant cannabis use was the hippocampus (HC); a study by Edrup et al., reported a reduction in bilateral HC volumes, while a similar study found alterations in both volume and shape [29,30]. Other findings of interest included larger areas of deflation in the left HC in schizophrenic patients with cannabis use when compared to healthy control groups [20]. Other authors found cannabis-related HC shape differences that correlated to deficits in episodic memory performance [31]. On the other hand, two studies reached opposite conclusions; one study found no significant differences when comparing first-episode schizophrenia patients with and without cannabis use with regard to hippocampal density [28], while another showed larger left and right HC volumes in adolescents with SZ, but only in those who consumed cannabis [32].

There were also changes evidenced in the size of the amygdala in cannabis users with SZ. A study found a significant effect of cannabis use on schizophrenic cannabis users, which was further altered with antipsychotic medication, with a smaller amygdala when compared to non-users [43]. This falls in line with another study’s result that pointed to a reduction in volume in the amygdala of SZ patients with and without cannabis use [44]. However, when looking at thalamic volume, an opposite association appears, with larger grey matter density evidenced in the left and right thalamus [44]. These results are similar to other studies, where the right thalamus was bigger in SZ patients with concomitant cannabis use when compared to healthy controls (HCs) [32,46]. Furthermore, a study that used sparse canonical correlation analyses found a modest increase in subcortical volumes of the basal ganglia, particularly of the caudate, putamen, and pallidum—this change was moderately correlated with cannabis use (r = 0.23) [47]. In this study, global brain and cortical thickness were also negatively correlated with antipsychotic dosage [47].

#### 3.3.2. Cannabis and Connectivity Imaging Studies (fMRI and DWI)

Studies looking at brain connectivity in SZ patients with cannabis use disorder have assessed microstructural white matter connectivity (i.e., diffusion-weighted imaging studies) and functional connectivity (i.e., functional MRI). When looking at diffusion-weighted imaging (DWI) and fractional anisotropy (FA), there is a correlation between a younger age of onset in alcohol consumption and lower FA values in the left thalamic and left parahippocampal radiation and a relation between the duration of illness and full brain FA value; FA can be considered an index that showcases the integrity of white matter (i.e., structural connectivity) [16]. Another study found a reduced FA value in the left posterior corpus callosum in schizophrenia patients who had not consumed cannabis when compared to consumers with and without schizophrenia [22]. Yet another study found that the FA decrease was present when compared to HCs as well as patients’ siblings [23]. The same study states that, over time, cannabis consumers had a more pronounced decrease in their FA values compared to non-consumers [23]. Furthermore, consumers with schizophrenia showed a decrease in FA in the brainstem, internal capsule, corona radiata, and superior and inferior longitudinal fasciculus compared to non-consumers [13]. On another hand, FA values in the anterior internal capsule, fasciculus uncinatus, and frontal white matter were elevated in cannabis users compared to HCs and in schizophrenia patients with cannabis use compared to HCs [24]. A longitudinal study found that, even with a relatively short follow-up period (i.e., 18 months), significant changes were found between schizophrenia, cannabis user, and healthy control groups [25], with a decrease over time in cannabis users and an increase in patients with schizophrenia. Finally, with regard to white matter, a study found that in patients with a first episode of psychosis, there was a positive relationship between whole-brain white matter volume and exposure to cannabis [16].

Regarding connectivity mapping in these patients, an author found a decrease in connectivity between the nucleus accumbens and structures involved in reward circuits (i.e., the ventral anterior cingulate cortex, orbitofrontal cortex, prefrontal cortex, insular cortex, premotor cortex, and parahippocampal cortex) [38]; another study concluded that the medial prefrontal cortex also presented decreased activation in these patients [39]. However, when performing emotional memory testing, the left calcarine fissure, right fusiform gyrus, and right inferior frontal gyrus showed increased activation in comparison to control groups [42]. Additionally, a study looking at resting state fMRI found hyperconnectivity in patients compared to HCs between the prefrontal cortex and the precuneus, which was apparently reduced with the administration of cannabinoids [40]. Another study found, however, that cannabis use showed no effect on cognitive testing or connectivity mapping in patients [41]. Other studies explored the activation of the amygdala via fMRI. It was found that the use of clozapine and risperidone on patients with schizophrenia and cannabis consumption produced a greater activation in response to positive and neutral images in the amygdala [45]. In contrast, subjects with a diagnosis of schizophrenia and no cannabis consumption showed significantly reduced activation in the left amygdala [30].

#### 3.3.3. Smoking (Nicotine) and Volumetric/Morphometric MRI

Only one study looked at brain volumetry in SZ patients and nicotine abuse; they found a reduction in the volume of the cingulate and insular cortices in both SZ patients and HCs [57]. Additional research is necessary to ascertain whether the interaction between nicotine addiction and schizophrenia induces volumetric alterations in the brain; nevertheless, it seems that this is not the case. Nonetheless, connectivity seems to be impacted.

#### 3.3.4. Smoking (Nicotine) and Connectivity Imaging Studies (fMRI and DWI)

Cullen et al. (2012) found a reduced FA index in SZ patients in whole-brain white matter and an even lower index in smoking SZ patients when compared to other groups [55]. However, the same study stated that the IQ of patients also had a correlation to the FA index, which may act as a confounding factor [55]. These findings are supported by another study, which found a similar FA index in smoking SZ patients when compared to non-smokers [56].

Regarding fMRI findings, a study found decreased activation in bilateral midline frontal cortices in comparison to HCs [58]. The same group, in another study, demonstrated decreased resting state functional connectivity, especially in the dorsal anterior cingulate cortex right limbic circuit [59]. The latter results were supported by another study that analysed the intrinsic brain activity in the same type of patients and found reduced intrinsic brain activity in SZ smoker patients when compared to other groups and a negative correlation between this parameter and the severity of SZ symptoms [60]. In contrast to this group of studies, some regions were found to have higher activation. These include the right insula and cerebellum [61] and the head of the right caudate nucleus and ventromedial prefrontal cortex [62]. It was found that patients with SZ without nicotine use have higher connectivity between the nucleus accumbens and the middle temporal gyrus, as well as between the precuneus and lateral occipital cortex, when compared to HCs [63].

#### 3.3.5. Alcohol and Volumetric/Morphometric MRI

In patients with SZ and comorbid alcohol use, significantly smaller volumes of total grey and white matter were found; even in regression models, SZ remained a relevant factor for smaller white matter volumes and larger volumes of cerebrospinal fluid [64]. These results are supported by Mathalon et al. (2003), where similar grey matter volume deficits were found in the prefrontal and superior temporal gyri [65]; reductions in the cortical volume of the insula, medial, and dorsolateral frontal cortex, ventrolateral prefrontal cortex, and parieto-occipital cortex of alcohol-consuming SZ patients compared to non-SZ subjects has also been reported [66,67]. Additionally, subcortical structures also seem to be affected; the putamen, nucleus accumbens, and caudate nucleus were found to be smaller in SZ groups when compared to healthy individuals [68]. These findings match with those of Smith et al. (2011), which stated that SZ and alcohol use resulted in abnormalities of the shape of the hippocampus, thalamus, striatum, and globus pallidus [69]. Furthermore, reductions in the volume of the cerebellar vermis have also been found in SZ patients with alcohol abuse [70,71]. On the other hand, a study looking into participants with alcohol abuse alone or alcohol abuse with the abuse of at least one other drug showed no difference between these two groups or when compared to those without substance use history; brain abnormalities in this study were determined by a qualitative assessment of the MRIs [73].

#### 3.3.6. Alcohol and Connectivity Imaging Studies (fMRI and DWI)

Few of our included studies looked at functional or structural connectivity in SZ patients with alcohol abuse disorder. Higher activation in certain brain regions like Broca’s area, Wernicke’s area, the primary motor cortex, the temporal fusiform gyrus, and the anterior cingulate area has been reported in these patients [72]. Furthermore, a study using sparse canonical correlation analyses reported that alcohol use was negatively correlated with FA values (r = −0.15) in the thalamus, fornix, and corpus callosum (genu and body) [47].

#### 3.3.7. Stimulants (Cocaine/Amphetamines) and Structural/Functional MRI Studies

Only two articles reported neuroimaging findings in patients with SZ and concomitant stimulant drug use (i.e., cocaine, amphetamines). When comparing patients with SZ to patients with stimulant-induced psychosis, a smaller mean volume in the left planum temporale was reported in the latter [49]; furthermore, in those patients, lower FA values were also identified in subcortical structures and in the cerebral peduncles [49]. Finally, in another study looking into changes in magnetic resonance spectroscopy (MRS), early-phase schizophrenia methamphetamine users had a greater decrease in N-acetyl aspartate and glutamate in the medial prefrontal cortex when compared to first-episode psychosis patients [50].

#### 3.3.8. Cumulative Substance Abuse and Structural/Functional MRI Studies

Some studies analysed subjects with SZ and the cumulative use of various substances. The results showed that these patients had reduced phosphocholine, myoinositol, and phosphocreatine levels in MRS and increased bilateral striatal grey matter volume when compared to non-abusers and non-schizophrenic patients [51,52]. Another study showed that when compared to HCs, SZ patients had significantly higher activation in the left superior temporal gyrus, left superior parietal lobule, right supramarginal gyrus, and cerebellum (left tonsil and pyramis); furthermore, when looking at only SZ patients with past substance abuse, decreased white matter activation was found in the right inferior parietal lobule and both middle frontal gyri when compared to SZ patients without substance abuse [54]. In contrast, a study looking into 41 participants with recent psychosis and consumption of cannabis, stimulants, opiates, hallucinogens, and alcohol found no significant difference in volumetric measurements between consumers and non-consumers; this lack of association was maintained when using multivariate approaches, diagnostic subgroups, and severity of substance abuse [53].

### 3.4. Bipolar Disorder and Substance Use Disorder

#### 3.4.1. Cannabis and Volumetric/Morphometric MRI

When looking at volumetric studies in patients with bipolar disorder (BD) and cannabis abuse, decreased grey matter volume and cortical thickness were reported in the left and right fusiform gyri and in the right middle frontal gyrus [26,75]. Additionally, an increase in the grey matter volume in the right caudate nucleus and precentral gyrus has also been reported [75].

#### 3.4.2. Cannabis and Magnetic Resonance Spectroscopy (MRS)

When analysing metabolic changes in these patients through the means of MRS, increased levels of N-acetyl aspartate in the ventrolateral prefrontal cortex were reported in the cannabis use disorder group—significantly higher than the BD and BD/cannabis groups (*p* = 0.0002)—and were associated with the amount of cannabis consumed [74]. No differences were found in the levels of N-acetyl aspartate between BD patients and HCs [74].

#### 3.4.3. Smoking (Nicotine) and Volumetric/Morphometric MRI

Only one study looked at the association between nicotine abuse and BD; MRI results showed reduced cortical thickness of the left anterior cingulate cortex in smoking vs. non-smoking BD patients [57].

#### 3.4.4. Alcohol and Volumetric/Morphometric MRI

In a study that investigated the relationship between adolescent alcohol use disorders and mental disorders, it was found that, regardless of the mental disorder, the patients who debuted with alcohol use in adolescence had a decrease in prefrontal cortical, prefrontal white matter, and cerebrospinal fluid volumes by MRI volumetry, while thalamic volumes remained unaffected [76]. In another study, it was found that alcohol use disorder was associated with thinner cortical volumes in the left superior frontal gyrus, (medial) right superior frontal gyrus, right insula, and bilateral parieto-occipital region, as well as a larger left lateral ventricle volume [67]. Nery et al. support these findings in their study, where they showed a smaller cortical thickness in the prefrontal cortex and anterior cingulate cortex [77]. Yet another study found out that in subjects with a family history of bipolar disorder, a higher number of drinks per drinking day correlates with lower insular grey matter volume [78].

Brain volumetric changes were also analysed in a study looking at 25 BD patients with alcoholism and 8 patients with only BD; no significant differences were found when comparing neurocognitive testing and MRI findings [83]. However, the right hippocampus of the BD patients with alcoholism was significantly smaller (*p* = 0.015) [83]. Furthermore, a study looking at 30 adolescents diagnosed with BD and no substance abuse at baseline reported that lower grey matter volumes in the prefrontal, dorsolateral prefrontal, orbitofrontal, right prefrontal, temporal pole, and insular cortices were associated with the development of cannabis and alcohol use disorders at 6–8 years of follow-up based on the results of the CRAFFT interview [82].

#### 3.4.5. Alcohol and Magnetic Resonance Spectroscopy (MRS)

When looking at neurometabolites with MRS, BD patients without alcohol use had higher levels of glutamate/glutamine in the left dorsolateral prefrontal cortex when compared to alcohol consumers; however, this significance was only found in male participants [79]. Another study showed further glutamate alterations, as well as GABA-related differences, in the anterior cingulate cortex, with a decrease in both these metabolites in BD patients with alcohol use [80]. When looking at other metabolites, a study analysing differences in glutathione (GSH) concentrations did not find a significant difference between HCs and BD patients; however, it did show a negative correlation between GSH levels and Alcohol Use Disorders’ Identification Test (AUDIT) scores in the hippocampus and dorsal anterior cingulate cortex [81].

#### 3.4.6. Unspecified Substance Abuse and Structural MRI and PET Scan

An Italian study looking at polyabusers (abusers of more than one drug) included a total of 10 participants with BD and substance abuse, 17 participants with BD and without substance abuse, 16 participants with substance-induced psychosis (SIP), and 54 HCs subjected to volumetric MRI and 18FDG PET (18-Fluorodeoxyglucose Positron Emission Tomography). In patients with bipolar disorder (with and without substance abuse) and patients with SIP, grey matter volume was significantly decreased in the superior temporal gyrus when compared with HCs (*p* < 0.05) [85]. However, when comparing BD participants with and without substance abuse, those with substance abuse had a significant reduction in grey matter volume in the left parahippocampus, right thalamus, and left cerebellum (*p* < 0.005) [85]. In addition, when looking at BD participants’ metabolism in the PET scan, those with substance abuse had decreased grey matter metabolism in the superior and inferior temporal gyri bilaterally and increased grey matter metabolism in the right thalamus and right cerebellum when compared with HCs (*p* < 0.005); when compared to BD participants without substance abuse, the polyabusers had significantly decreased grey matter metabolism in the left posterior cingulate (*p* < 0.005) [85].

Another study, looking at 18 participants with manic and mixed BD and 15 HCs, included only five subjects with a concomitant substance use disorder; they looked at signal hyperintensities in MRI, reporting no significant difference between BD participants and healthy controls and between substance use and no use [84].

### 3.5. Depression and Substance Use Disorder

#### 3.5.1. Cannabis and Volumetric/Morphometric MRI

In relation to patients with depression and cannabis use, some structural brain changes were reported. Firstly, when studying the sulcogyral patterns of the right orbitofrontal cortex (i.e., a morphological variation of the OFC established in early life), a type III pattern was associated with higher cumulative lifetime cannabis use [86]. Furthermore, reduced cortical thickness in the medial orbitofrontal cortex and superior frontal cortex was found in depressive cannabis users [86]. Secondly, a study looking at 93 young adults with depression and/or cannabis use and 48 HCs reported that in cannabis users, reduced cortical thickness was detected in the medial orbitofrontal, middle temporal, and superior frontal regions [87]. Furthermore, it seems that the interaction of cannabis use and depression results in an additive effect on the cortical reduction in the middle temporal gyrus [87].

#### 3.5.2. Cannabis and Functional MRI

Other studies looking at the effect of cannabis use on the brain’s functional connectivity of depressed patients have found differences in the connectivity of the right caudate/temporal gyrus/parahippocampal gyrus circuit and right medial frontal gyrus and an additive effect in the alteration of connections in the left culmen/fusiform gyrus area [88]. Additionally, connectivity between the default mode network and the right and left temporal, occipital, and fusiform cortices, right precuneus and culmen, right ACC, and left superior frontal gyrus was reduced in long-term depressive cannabis users [88,89]. In another study, patients with depression presented higher activation in the left supramarginal gyrus when compared to either comorbid or HCs [90]. Yet, another study showed that the activation and reactivity of the amygdala in depressive patients were significantly correlated to the frequency of cannabis use, with a reduction in cannabis use producing an increase in the amygdala’s reactivity [91].

#### 3.5.3. Smoking (Nicotine) and Volumetric/Functional MRI

Patients with depression alone showed increased left hemisphere cortical thickness compared to depressive smokers and non-smoking controls, with no significant differences when comparing cumulative depressive subjects and healthy controls or when taking into account only depressive smoker patients vs. healthy controls [94]. In contrast, fMRI analysis showed a relationship between depressive symptoms (i.e., Hamilton Depression Rating Scale scores) and neural activity, as well as post-smoking BOLD activation, in the frontal gyrus, superior temporal gyrus, hippocampus, and anterior cingulate [95].

#### 3.5.4. Alcohol and Volumetric/Functional MRI

Patients with depression and alcohol use disorder showed a significantly greater grey matter volume in the overall cortical volume as well as in the lateral occipital cortex, medial orbitofrontal cortex, middle temporal cortex, and isthmus of the cingulate cortex in volumetric MRI compared to alcohol-dependent users without depression [92]. Another study showed an increase in prefrontal cerebrospinal fluid in subjects with early-onset consumption of alcohol with other psychiatric comorbidities; however, it should be noted that while the most common comorbidity was depression, this study’s authors included other psychiatric disorders in their analyses [76].

In contrast, fMRI studies reported hyperactivity of the thalamus and putamen in depressive alcohol users when subjected to a stop signal task (i.e., subjects are presented with an imperative stimulus and are asked to respond as fast as possible [105]) [93]; although there was no significant difference in response inhibition between the studied groups [93].

#### 3.5.5. Opioids and Structural/Functional Connectivity in MRI

A study looking at the diffusivity of white matter tracts in 26 male past heroin users and 32 HCs found no association between the changes in the levels of fractional anisotropy or mean diffusivity and the depression levels in heroin users; however, anxiety levels in heroin users changed as the level of mean diffusivity in the uncinate fasciculus varied (*p* = 0.022, r2 = 0.34) [97]. Another study looking at fMRI on a 7T scanner demonstrated lower activation in heroin users with depression when compared to HCs in the following areas during the anticipation phase of a reward task: right superior frontal gyrus, supplementary motor area, paracingulate gyrus, frontal pole, precuneus, caudate, thalamus, posterior cingulate gyrus, left precentral gyrus, anterior cingulate cortex, middle frontal gyrus, and inferior frontal gyrus [98].

#### 3.5.6. Ketamine and Functional MRI

Only one study looked at chronic ketamine use in patients with depression, finding a higher depression score (in the Center for Epidemiologic Studies Depression Scale [CES-D]) in females when compared to HCs [96]. Furthermore, when looking at resting-state fMRI, a negative correlation was found in the connectivity between the subgenual anterior cingulate cortex and the orbitofrontal cortex (i.e., the higher the CES-D score, the less connectivity between these two areas) [96]. Also, positive correlations were found in the connectivity between the subgenual anterior cingulate cortex and (1) the bilateral superior temporal gyrus in male chronic ketamine users and (2) the dorsomedial prefrontal cortex in female chronic ketamine users [96].

#### 3.5.7. Unspecified Substance Abuse and Functional MRI

Only one study analysed unspecified substance abuse disorder in patients with depression; however, this study included both depression and PTSD in the same group [99]. By using resting-state fMRI, they found that female participants exposed to childhood maltreatment who later developed both substance abuse and a comorbid psychiatric disorder (depression/PTSD) have increased functional connectivity between the right rostral anterior cingulate cortex/medial prefrontal cortex and the left temporal–parietal junction [99].

### 3.6. Anxiety and Substance Use Disorder

#### 3.6.1. Alcohol and Volumetric/Functional MRI

When looking at volumetric studies, greater volumes of grey matter were found in the lateral occipital cortex, medial orbitofrontal cortex, middle temporal cortex, and the isthmus of the cingulate cortex [92]. In contrast, another study stated that comorbid patients showed a decrease in prefrontal cortical grey and white matter and cerebrospinal fluid volumes [76]. Certainly, when looking closely at white matter, one study using diffusion-weighted MRI found higher fractional anisotropy in the corpus callosum, internal capsule, corona radiata, superior parietal, lateral occipital, and posterior cingulate [102].

Finally, in patients with alcohol use and anxiety, fMRI studies have shown higher activation in the right superior frontal gyrus, middle frontal gyrus, inferior frontal gyrus, precentral gyrus, thalamus, putamen, and insula when compared to HCs [93,100,101].

#### 3.6.2. Heroin and Diffusion-Weighted MRI

Heroin use is related to higher levels of anxiety and depression because of the impact on the emotional regulatory system. The use of DWI imaging to determine differences in this aspect has shown that diffusivity changes in the left uncinate fasciculus strongly relate to anxiety and prolonged heroin use; the same association was not found with depression [97].

### 3.7. Post-Traumatic Stress Disorder (PTSD) and Substance Use Disorder

#### Alcohol and Functional MRI

We only found two studies analysing PTSD subjects with comorbid alcohol use disorder and neuroimaging. The first one showed that PTSD subjects with alcohol use had higher activation in the parahippocampal gyrus compared to non-comorbid subjects [103]. On the other hand, the second article showed that patients with alcohol use had reduced activity in hippocampal regions and in the anterior cingulate cortex [104]. Another study looking at unspecified substance abuse found an increase in functional connectivity between the right rostral anterior cingulate cortex, the medial prefrontal cortex, and the left temporal–parietal junction [99].

### 3.8. Key Findings

The present review involves diverse study types, imaging methods, and brain areas/neurometabolites being analysed. While there are various brain areas affected, it is evident that key regions of interest are constantly affected in diverse disorders and with the abuse of different substances. Areas related to reward processing (prefrontal cortex, striatum, and thalamus) have alterations in volumetric and connectivity-related studies. The same is true for information-processing areas, including the frontal and prefrontal cortices, the hippocampus, and other important areas such as the phonological loop (i.e., the frontal and temporal gyri and the parietal cortex). Another functional circuit that is constantly affected is emotional and memory-related areas (the insular cortex, hippocampus, amygdala, and prefrontal cortex). A concise summary of the most common alterations found in our review can be found in Table 2.

Other common findings include a volume decrease in these key areas, as well as a decrease in connectivity and activation in information-processing areas, evidenced by a decrease in FA, resting-state activation, and a DTI-evidenced decrease in connectivity. Increases in the activity and connectivity of emotion-related areas (i.e., the thalamus, temporal gyri, parahippocampal areas, and the amygdala) are also evidenced in depression, anxiety, PTSD, and schizophrenia. There are also clear differences between patients with psychiatric disorders and those with the same conditions who also suffer from substance abuse. It is interesting to note that in various studies, comorbid groups have alterations in different brain areas and metabolites than non-consumers or consumers without other psychiatric conditions.

## 4. Discussion

To the best of our knowledge, this is the first study thus far that has comprehensively reviewed MRI-evidenced changes in the brains of patients with psychiatric disorders and comorbid substance abuse. After reviewing the 92 included studies, we found that the majority of these focused on schizophrenia (64%), whereas other disorders (i.e., bipolar disorder, depressive disorder, anxiety disorder, and PTSD) were not as frequently studied with regard to comorbid substance abuse and neuroimaging changes. Additionally, most of the included studies relied heavily on volumetric MRI analysis or BOLD signal analysis (fMRI), whereas other techniques such as magnetic spectroscopy (MRS) or diffusion-weighted imaging (DWI) were somewhat neglected (Table 3).

### 4.1. Findings in Schizophrenia

The specific characteristics of the included articles looking into schizophrenia, substance use, and neuroimaging changes can be found in Table S1 of the Supplementary Materials.

Regarding patients with schizophrenia, it was determined that most of them had decreased grey or white matter in various cortical and subcortical areas. Most of the findings in MRI and fMRI techniques indicated a decrease in grey matter density and volume [15,18,21,27] and white matter integrity [20,22,23] and activity [40] in cortical areas. This falls in line with long-standing evidence of progressive structural brain changes in these patients [106].

On the other hand, the use of substances produced further changes in patients with schizophrenia. Cannabis use in adolescents with schizophrenia was associated with larger brain volumes, specifically of the parietal and frontal lobes [32], and with a decrease in the prefrontal cortex volume [33] and the amygdala [43,44]. It has been shown that cannabinoid receptors are more prevalent in the amygdala, neocortex, and memory-related brain structures [107], which would explain why structural changes occur more frequently in these regions. Furthermore, both cannabis use and schizophrenia are related to long-term memory and cognitive dysfunction [108,109]. Patients with schizophrenia show a decreased prefrontal cortex size as well as an increase in activation in reward-related circuitry [110], which would explain why these patients show an affinity for cannabis (and other substance) abuse.

In patients with schizophrenia and alcohol abuse, there was increased activity identified by the fMRI in the motor cortex, anterior cingulate area, fusiform gyrus, and Wernicke’s area [72] as well as decreased activity in the ventrolateral prefrontal cortex [66]. Additionally, smaller volumes in total grey and white matter [64], vermian areas [71,72], medial and dorsolateral frontal and parietal cortices [67], and the temporal lobe [65] were found. Other regions like the hippocampus, thalamus, striatum, and globus pallidus were shown to have volumetric and morphologic alterations [69]. The use of alcohol has been shown to produce both short- and long-term neuropathic changes, both from direct neurotoxicity and concomitant nutritional deficiency [111]. Additionally, ethanol interacts with various specific neurotransmitter receptors, including the inhibition of NMDA receptors and GABA, dopamine, and serotonin excitation, producing both cortical and subcortical neurodegeneration [112]. The findings of this review are in line with previous evidence, seeing as [historically] empirical evidence suggests that the same neurotransmitters and related circuits are affected by schizophrenia [113].

When looking into nicotine use, patients with schizophrenia showed changes in their brain structure in both cortical and subcortical areas. There were significant differences in the volume of the medial prefrontal cortex, the bilateral caudate, and the postcentral and superior temporal gyrus [60] and increased activity in the nucleus accumbens [63] and the caudate nucleus [62]. Cortical structures had reductions in volumes of grey [57] and white matter [55]. fMRI showed decreased activation of frontal cortices [58] as well as alterations in subcortical structures. Decreased resting state connectivity in the limbic circuit was found [59], as well as higher activation in the insula and cerebellum [61]. DWI studies found that the left anterior thalamic radiation was smaller than in control groups when compared to the schizophrenia/nicotine user group [56]. The use of nicotine has been known to cause alterations in the morphology and blood flow of the brain [114]. Additionally, nicotine disturbs various neurotransmitters and brain circuits, as shown in studies using rat models [115]. These characteristics of nicotine abuse support the findings of our review, considering that most of the affected areas are richly vascularized cortical and subcortical structures involved with dopamine and norepinephrine, as well as other neurotransmitters that are affected by both schizophrenia and nicotine use [114,116].

In patients with schizophrenia and substance abuse, some systems such as the glutaminergic and dopaminergic networks are altered, especially affecting the nucleus accumbens [110]. Other studies have found decreased activity in the left ventral striatum, which is associated with the reward system; this deactivation has been shown to be correlated with the severity of negative symptoms and the craving intensity for substances such as alcohol [117]. On another hand, it has been shown that in patients with a genetic predisposition to schizophrenia, developmental alterations in certain brain structures such as the hippocampus, prefrontal cortex, and nucleus accumbens may cause a motivational deficit like that experienced in people with substance use [118]. In consequence, the effects of drugs will be of greater intensity in these patients due to alterations in the dopaminergic system, increasing the likelihood and maintenance of drug use [118].

### 4.2. Findings in Bipolar Disorder

The specific characteristics of the included articles looking into bipolar disorder, substance use, and neuroimaging changes can be found in Table S2 of the Supplementary Materials.

As with patients with schizophrenia, subjects with bipolar disorder have been shown to have a higher risk of developing substance abuse disorder [119], with neuroanatomical and functional brain changes. Regarding cannabis use, volumetric reductions were found in the ventrolateral prefrontal and anterior cingulate cortices [74], as well as a lower cortical density in the frontal, fusiform, and superior gyri [26,75]. When analysing functional imaging results in these patients, there was lower activation of the amygdala, nucleus accumbens, and thalamus [74].

In patients with bipolar disorder and alcohol use, there is evidence of changes in cortical surfaces. These patients show a decrease in prefrontal cortical and prefrontal white matter [76] and lower grey matter volume in rostral and dorsal prefrontal cortex, anterior cingulate, and parieto-occipital regions [67,78,79,82]. In MRS studies, it was found that patients had lower levels of glutamine, glutamic acid, and GABA in the left dlPFC and the dorsal ACC [79,80]. In patients with nicotine abuse, it was found that cortical thickness is reduced in smoking patients, especially in the left anterior rostral cingulate cortex and the left insular cortex [57].

There are several studies that link bipolar disorder to the development of a substance use disorder. An epidemiological study showed that, compared to subjects with other axis I disorders, individuals with bipolar I disorder had the highest lifetime rates of alcohol use disorders (46% of patients with BD) and drug use disorders (41% of patients) [120]. On a physiopathological level, the results reviewed in our study fall in line with other authors. For example, a systematic review of neuroimaging findings in patients with early-onset bipolar disorder also found volumetric reductions in the superior temporal gyrus, putamen, thalamus, amygdala, and hippocampus, as well as in cortical grey matter [121]. Additionally, alterations in the structure of the amygdala have been known to be related to the development of bipolar disorder [122]. Another link between the two would be the presence of common clinical characteristics, such as the presence of impulsive behaviour, and genetic and epigenetic alterations [123]. The comparison of these studies with our findings suggests that alterations of cerebral structures are due to the presence of bipolar disorder and are aggravated by comorbid substance abuse.

### 4.3. Findings in Depressive Disorder

The specific characteristics of the included articles looking into depressive disorder, substance use, and neuroimaging changes can be found in Table S3 of the Supplementary Materials.

Differences are also found in patients with depressive disorder and cannabis use in cortical and subcortical brain structures. In MRI studies, a reduced cortical thickness in the orbitofrontal cortex, temporal gyrus, and frontal cortex was found [87], as well as decreased prefrontal cortical and white matter volumes [76]. In fMRI studies, decreased amygdala activation [91] and higher activation in the supramarginal gyrus [90] and in areas of motor planning, motor control, and reward processing [88] were found in comorbid patients. In patients with alcohol abuse, higher volumes of overall grey matter have been found [92]. Regarding functional activity, increased activation of the thalamus and putamen has been reported [93], whereas nicotine users showed reduced activity in the mesocorticolimbic system [95] and a volume reduction in the postcentral and temporal cortex [94].

The orbitofrontal cortex, anterior cingulate, and amygdala are theorized to be important components of the reward and motivational circuits of the brain, depending mainly on serotonin, dopamine, and GABA as principal neurotransmitters [124]. Depression, as well as substance abuse, both have an important component of altered reward circuitry, with serotoninergic neuronal pathways being the more prominent of these [125]. Furthermore, substance abuse has been shown to alter the circadian rhythm of patients [126], the latter being an important regulator of serotoninergic metabolism [127].

### 4.4. Findings in Anxiety Disorder

The specific characteristics of the included articles looking into anxiety disorder, substance use, and neuroimaging changes can be found in Table S4 of the Supplementary Materials.

Patients with anxiety disorder and alcohol use showed a reduction in frontal grey and white matter [76]. Different cortical areas showed greater volumes and activation, like the right frontal lobe [100], as well as increased total white matter [102] in the lateral occipital cortex, orbitofrontal cortex, and cingulate cortex [92]. Likewise, patients showed increased activation in the thalamus and putamen [93] and augmented inter-hemispheric insular connectivity [101].

As with the psychiatric disorders discussed before, there is an epidemiological correlation between anxiety and substance use disorder [128]. Substances are used by patients with anxiety as a short-term escape mechanism from psychosocial stress and serve as ‘sedatives,’ even in animal models [129,130]. However, it is known that the prolonged use of substances worsens anxiety symptoms and anxiety disorders are related to lifetime alcohol consumption [131,132]. These theories fall in line with neurophysiological alterations in the central, medial, and basolateral nuclei of the amygdala, which are involved in the regulation of emotions, play a part in the development of anxiety, and cause the dysregulation of GABAergic circuits [133]. Subsequently, alcohol also stimulates GABA-secreting neurons [134], perpetuating alcohol consumption in patients with anxiety disorder.

### 4.5. Findings in Post-Traumatic Stress Disorder

The specific characteristics of the included articles looking into post-traumatic stress disorder, substance use, and neuroimaging changes can be found in Table S5 of the Supplementary Materials.

With regard to patients with PTSD and substance use, we found a decrease in prefrontal cortical and prefrontal white matter in alcohol users [76]. In the same type of patients, a positive activation in the parahippocampal region was evidenced [103], as well as reduced activity in the hippocampus [104]. While there is scarce evidence regarding PTSD patients with other types of substance abuse, PTSD and anxiety disorder have a similar pathogenesis and clinical course [135]. This theory is supported by multimodal neuroimaging results in PTSD, where similar circuitry between the prefrontal cortex, hippocampus, and amygdala is involved [136]. Thus, it stands to reason that the same comorbid pathogenesis between PTSD and alcohol abuse is produced, with the same alteration of amygdalar circuitry and GABA-related alterations [133,134,136].

### 4.6. Limitations

The topic of this study implies a considerable breadth of scope. One of the main limitations is that most studies have a small sample size, which in turn hinders the possibility of having a homogenous sample for analysis and comparison; therefore, the results tend to have various confounding factors. While most studies do control for secondary variables, like age, sex, left-or-righthandedness, and comorbidities, there are other cofounders that are difficult to control for, like previous pharmacological treatment, that could muddle potential findings, as the chronic use of psychiatric medication has been proven to significantly affect the neurochemistry and architecture of the brain; therefore, patients receiving chronic or acute psychiatric treatment and treatment-naïve patients may have different profiles of neurometabolites or structural changes in the brain and should be further assessed in future studies by correctly segmenting and differentiating these populations. Another limiting factor is the scarce amount of evidence regarding certain psychiatric disorders and neuroimaging applications. Of the studies yielded by our search, most studied schizophrenic patients with cannabis use, while disorders like PTSD and substances like stimulants were rarely reported. Similar limitations were found regarding neuroimaging modalities; MR spectroscopy or diffusion-tensor imaging modalities were not as frequent as structural MR imaging or functional MR imaging.

This heterogeneity of study types, as well as the absence of a standardized protocol in the included articles for reviewing this type of patients (i.e., having patients with different or no treatments, not determining doses of substance consumption, etc.), is an important limitation of the current review. While some of our main objectives were to determine key brain areas affected by various disorders and to scope potential diagnostic methodologies for these disorders, the absence of current literature deepening our understanding of the neurochemistry involved in different psychiatric disorders and substance abuse is, for the moment, a severe limitation to achieving these objectives. New research and trials should be focused on the application of these study types (i.e., magnetic resonance spectroscopy, diffusion tensor imaging, and diffusion-weighted MR) to further our understanding of the neurochemistry of psychiatric disorders and substance abuse, as well as to determine key components in the development of these conditions, to develop newer and more precise diagnostic methods for clinical application in the field of neuropsychiatry.

Regarding the review process, the main limitation was the nature of the data and the number of studies. Since our search yielded mostly studies of an observational nature with a high degree of heterogeneity, the process did not allow the use of meta-analysis, which limits the potential comparability of raw data. However, to the best of our knowledge, this is the first systematic review that compiles the results of neuroimaging findings from axis I patients with comorbid substance abuse. During this endeavour, we have identified some future avenues of improvement regarding research in this area; first, larger sample sizes are desirable in order to increase the robustness of neuroimaging findings; second, subgroup analysis should be conducted looking at groups afflicted with psychiatric disorders without previous treatment or in a relatively early phase of their condition, so that brain structure is unaffected by potential pharmacological bias; third, the standardization of different parameters in neuroimaging studies is necessary, including the standardization of patient characteristics (i.e., the duration and type of treatment, the frequency of substance consumption, and controlling for substances other than the main subject of study) as well as study parameters (i.e., the units of order and metabolites analysed in MRS, stimuli used in fMRI protocols, units in MRI volumetric studies).

## 5. Conclusions

The present study gives a comprehensive overlook of the main neuroradiological (structural and functional) findings in patients with psychiatric disorders and comorbid substance abuse. We synthesize results in subjects with bipolar disorder, schizophrenic disorder, major depressive disorder, anxiety disorder, and PTSD with the use of cannabis, nicotine, alcohol, opioids, and stimulants. Notwithstanding the limitations of this review (i.e., small sample sizes and few studies reviewing certain substances or disorders), there exists evidence of key brain structures showing alterations, mainly those that have a role in cognitive processing and reward circuitry. We hope that this study sets the groundwork for future research on the subject as well as new studies that focus on people with substance abuse and psychiatric conditions, who are often overlooked in neuroimaging research. The findings reported here show implications for the current understanding and classification of psychiatric disorders, showing that there is an important neurological component that is affected in psychiatric patients and drug users. Additionally, the findings presented here may indicate potential targets for pharmacological therapies in future clinical trials for drug addiction and psychiatric comorbidities.

## Figures and Tables

**Table 1:** Newcastle–Ottawa grading scale showcasing the bias assessment of each study.

Reference	Author	Year	Selection	Comparability	Exposure/Outcome
[12]	Abush	2018	****	**	***
[13]	James	2011	****	**	***
[14]	Schiffer	2010	****	**	***
[15]	Cohen	2012	****	**	***
[16]	Cookey	2018	***	**	**
[17]	Quinn	2018	****	*	***
[18]	Rapp	2013	****	**	***
[19]	Solowij	2011	****	**	***
[20]	Solowij	2013	****	**	***
[21]	Rais	2008	****	**	***
[22]	Dekker	2010	****	**	***
[23]	Domen	2019	****	**	***
[24]	Peters	2009	***	**	***
[25]	Epstein	2015	****	**	***
[26]	Hartberg	2018	****	**	***
[27]	Rais	2010	****	**	***
[28]	Bangalore	2008	****	**	***
[29]	Ebdrup	2010	****	**	***
[30]	Malchow	2013	****	**	***
[31]	Smith	2015	****	**	***
[32]	Kumra	2012	****	**	***
[33]	Cunha	2013	****	**	***
[34]	Szeszko	2007	****	**	***
[35]	Epstein	2015	****	**	***
[36]	Epstein	2014	****	**	***
[37]	Jernigan	1991	****	**	***
[38]	Fischer	2014	****	**	***
[39]	Rigucci	2018	****	**	***
[40]	Whitfield-Gabrieli	2018	****	**	***
[41]	Peeters	2015	****	**	***
[42]	Bourque	2013	****	**	***
[43]	Buchy	2016	****	**	***
[44]	Koenders	2015	****	**	***
[45]	Machielsen	2018	****	**	***
[46]	Smith	2014	****	**	***
[47]	Haller	2013	****	**	***
[48]	Moser	2018	****	**	***
[49]	Alexander	2019	****	**	***
[50]	Crocker	2014	****	**	***
[51]	Bernier	2016	****	**	***
[52]	Potvin	2007	****	**	***
[53]	Wobrock	2009	**	**	***
[54]	Wojtalik	2014	****	**	***
[55]	Cullen	2012	****	**	***
[56]	Zhang	2010	****	*	***
[57]	Jørgensen	2015	****	**	***
[58]	Moran	2018	****	**	***
[59]	Moran	2013	****	**	***
[60]	Liu	2018	****	**	***
[61]	Postma	2006	****	**	***
[62]	Potvin	2016	****	**	***
[63]	Potvin	2019	****	**	***
[64]	Nesvag	2007	****	**	***
[65]	Mathalon	2003	****	**	***
[66]	Gizewski	2018	****	**	***
[67]	Lange	2017	****	**	***
[68]	Deshmukh	2005	****	**	***
[69]	Smith	2011	****	**	***
[70]	Joyal	2004	****	**	***
[71]	Sullivan	2000	****	**	***
[72]	Joyal	2007	***	*	**
[73]	Scheller-Gilkey	1999	****	**	***
[74]	Bitter	2014	****	**	***
[75]	Jarvis	2008	****	**	***
[76]	De Bellis	2005	****	**	***
[77]	Nery	2011	****	**	***
[78]	Kirsch	2021	****	**	***
[79]	Nery	2010	****	**	***
[80]	Prisciandaro	2017	****	**	***
[81]	Chitty	2014	****	**	***
[82]	Lippard	2017	****	*	***
[83]	Shad	2015	****	**	***
[84]	Strakowski	1993	****	**	***
[85]	Altamura	2016	****	**	***
[86]	Chye	2017	****	**	***
[87]	Radoman	2019	****	**	***
[88]	Osuch	2016	****	**	***
[89]	Thayer	2019	****	**	***
[90]	Nichols	2021	****	**	***
[91]	Cornelius	2010	****	**	***
[92]	Uhlmann	2019	****	*	***
[93]	Sjoerds	2014	****	**	***
[94]	Zorlu	2017	****	**	***
[95]	Kushnir	2013	****	**	***
[96]	Li	2017	****	**	***
[97]	Wong	2015	****	**	***
[98]	Yi	2019	****	**	***
[99]	Martins	2018	****	**	***
[100]	Karch	2008	****	**	***
[101]	Viswanath	2015	**	**	*
[102]	Kim	2016	****	**	***
[103]	Harlé	2020	****	*	**
[104]	Schuff	2008	****	**	***

**Table 2:** Key findings of the most relevant disorders and substances.

Disorder Type	Substance	MRI Study Type	Regions of Interest	Findings
**Schizophrenia**	Cannabis	Volumetry	TBV *	Reduction in TB *, GM *, and WM * volumes
			Cortical areas (left prefrontal, middle frontal, right fusiform, left superior gyrus, right supplementary motor cortex, inferior frontal, superior temporal, angular cortex, and parietal)	Reduction in total cortex volume
			Amygdala, hippocampus, cerebellum	Reduction in TB * and GM * volume
			Thalamus	Increased volume
		Connectivity	Left thalamic radiation, left parahippocampal radiation, brainstem, internal capsule, corona radiata, superior and inferior longitudinal fasciculus; connectivity between nucleus accumbens and structures involved in reward circuits	Reduction in FA *
			Left posterior corpus callosum, anterior internal capsule, fasciculus uncinatus, and frontal white matter; connectivity between prefrontal cortex and the precuneus	Increase in FA *
	Nicotine	Volumetry	Cingular and insulate cortices	Total volume reduction
		Connectivity	TB-WM *	Reduction in FA *
			Bilateral midline frontal cortices	Decreased activation
			Right insula, cerebellum, right caudate nucleus, ventromedial PFC *.	Increased activation
	Alcohol	Volumetry	TBV *	TBV * reduction
			Insula, medial and dorsolateral frontal cortex, ventrolateral prefrontal cortex, and parieto-occipital cortex; putamen, nucleus accumbens, and caudate nucleus; hippocampus, thalamus, striatum and globus pallidus; cerebellar vermis	Volume reduction
		Connectivity	Broca’s area, Wernicke’s area, primary motor cortex, temporal fusiform gyrus, and anterior cingulate area	Increased activation
	Stimulants	Volumetry	Left planum temporale	Reduced volume
		Connectivity	Subcortical structures and cerebral peduncles	Reduction in FA *
		Spectroscopy	Medial PFC *	Reduction in NAA * and glutamate levels
**Bipolar disorder**	Cannabis	Volumetry	Left and right fusiform gyri, right middle frontal gyrus	GM * decrease
			Right caudate nucleus and precentral gyrus	GM * increase
		Spectroscopy	Ventrolateral PFC	NAA * increase
	Nicotine	Volumetry	Left ACC	Decreased cortical thickness
	Alcohol	Volumetry	Prefrontal area; left superior frontal gyrus, right superior frontal gyrus, right insula, bilateral parieto-occipital cortices; cingulate cortex; hippocampus	Decreased GM * and WM *
			CSF * volumes	Decreased
			Ventricle volumes	Increased
		Spectroscopy	Left dorsolateral prefrontal cortex	Increased glutamate/glutamine
			ACC *	Decreased glutamate; decreased GABA
**Depression**	Cannabis	Volumetry	Medial orbitofrontal cortex and superior frontal cortex	Decreased cortical thickness
		Connectivity	Right caudate/temporal gyrus/parahippocampal gyrus circuit, right medial frontal gyrus, left culmen/fusiform gyrus area; right and left temporal, occipital, and fusiform cortices, right precuneus and culmen, right ACC *, and left superior frontal gyrus	Decreased connectivity
			Left supramarginal gyrus	Increased connectivity
			Amygdala	Increased activation with less cannabis consumption
	Nicotine	Volumetry	Left hemisphere cortical thickness	Decreased volume in depressive smokers
		Connectivity	Frontal gyrus, superior temporal gyrus, hippocampus, and anterior cingulate	Increased activation after smoking
	Alcohol	Volumetry	Overall cortical volume as well as in the lateral occipital cortex, medial orbitofrontal cortex, middle temporal cortex, and isthmus of the cingulate cortex	Increased GM * volume
		Connectivity	Thalamus and putamen	Increased activation
	Opioids	Connectivity	Right superior frontal gyrus, supplementary motor area, paracingulate gyrus, frontal pole, precuneus, caudate, thalamus, posterior cingulate gyrus, left precentral gyrus, anterior cingulate cortex, middle frontal gyrus, and inferior frontal gyrus	Decreased activation
	Ketamine	Connectivity	Subgenual anterior cingulate cortex and the orbitofrontal cortex	Decreased connectivity
			Subgenual anterior cingulate cortex and bilateral superior temporal gyrus or dorsomedial prefrontal cortex	Increased connectivity
**Anxiety**	Alcohol	Volumetry	Lateral occipital cortex, medial orbitofrontal cortex, middle temporal cortex, isthmus of the cingulate cortex	Increased volume
			Prefrontal cortical grey and white matter and cerebrospinal fluid volumes	Decreased volume in depressive smokers
		Connectivity	Corpus callosum, internal capsule, corona radiata, superior parietal, lateral occipital, and posterior cingulate	Increased FA *
			Right superior frontal gyrus, middle frontal gyrus, inferior frontal gyrus, precentral gyrus, thalamus, putamen, and insula	Increased activation
	Heroin	Connectivity	Left uncinate fasciculus	Increased diffusivity
**PTSD**	Alcohol	Connectivity	Parahippocampal gyrus	Increased activation
			Hippocampus, anterior cingulate cortex	Decreased activation

* Abbreviations: ACC: anterior cingulate cortex; CSF: cerebrospinal fluid; FA: fractional anisotropy; GM: grey matter; NAA: N-acetyl aspartate; PFC: prefrontal cortex; TB: total brain; TBV: total brain volume; WM: white matter.

**Table 3:** Number of articles that use different MRI-derived techniques.

Imaging Study ^1^	Articles Included	%
Structural MRI	55	52
HMRS	9	9
FMRI	28	26
DWI	14	13
TOTAL	106	100

^1^ Some articles use more than one study type on the same population. MRI: magnetic resonance disorder. HMRS: hydrogen-based magnetic resonance spectroscopy. FMRI: functional magnetic resonance imaging. DWI: diffusor-weighted imaging.

## Data Availability

All data are available in the manuscript.

## Supplementary Materials

The following supporting information can be downloaded at: https://www.mdpi.com/article/10.3390/jcm14072156/s1, Table S1: Included studies looking at schizophrenia, substance abuse, and neuroimaging changes.; Table S2: Included studies looking at bipolar disorder, substance abuse, and neuroimaging changes.; Table S3: Included studies looking at depressive disorder, substance abuse, and neuroimaging changes.; Table S4: Included studies looking at anxiety disorder, substance abuse, and neuroimaging changes.; Table S5: Included studies looking at anxiety disorder, substance abuse, and neuroimaging changes.
