# Supplementary material for: Axis I Psychiatric Disorders and Substance Abuse: A Systematic Review of Neuroimaging Findings
Source: J Clin Med. 2025 Mar 21;14(7):2156. doi: 10.3390/jcm14072156 (PMC11989531; doi:10.3390/jcm14072156)
Supplement: Supplementary file 1 [file jcm-14-02156-s001.zip › jcm-3502021-supplementary.pdf]

SUPPLEMENTARY FILE 1: SEARCH FORMULA FOR EACH DATABASE

Search Strategy

- **PubMed (MEDLINE):** (("Neuroimaging"[Mesh]) OR ("Magnetic Resonance Imaging"[Mesh]) OR ("Magnetic Resonance Spectroscopy"[Mesh]) OR MRI OR functional MRI OR MRS OR MR spectroscopy OR multimodal MRI) AND (("Feeding and Eating Disorders"[Mesh]) OR ("Mood Disorders"[Mesh]) OR ("Anxiety Disorders"[Mesh]) OR ("Bipolar and Related Disorders"[Mesh]) OR ("Schizophrenia Spectrum and Other Psychotic Disorders"[Mesh]) OR Axis 1 disorders OR Axis I disorders OR anxiety disorder OR panic attacks OR depression) AND (("Substance-Related Disorders"[Mesh]) or substance use OR substance abuse OR drug abuse).
- **Cochrane Controlled Register of Trials (CENTRAL):** ([mh "Neuroimaging"] OR [mh "Magnetic Resonance Imaging"] OR [mh "Magnetic Resonance Spectroscopy"] OR MRI OR functional MRI OR MRS OR MR spectroscopy OR multimodal MRI) AND ([mh "Feeding and Eating Disorders"] OR [mh "Mood Disorders"] OR [mh "Anxiety Disorders"] OR [mh "Bipolar and Related Disorders"] OR [mh "Schizophrenia Spectrum and Other Psychotic Disorders"] OR Axis 1 disorders OR Axis I disorders OR anxiety disorder OR panic attacks OR depression) AND (([mh "Substance-Related Disorders"]) or substance use OR substance abuse OR drug abuse).
- **Scopus:** ((Neuroimaging) OR (Magnetic resonance) OR (Magnetic*Resonance*Imag) OR (Functional*Magnetic*Resonance*Imag) OR ("Magnetic resonance spectroscopy") OR (MRI) OR (MRS) OR (FMRI) OR ("Multimodal Magnetic Resonance") OR (Diffusion MRI) OR ("Multimodal MR") OR ("MR Spectroscopy")) AND ((Feeding*and*eating*disorder*) OR ("eating disorder") OR (anorexia) OR (bulimia) OR (Mood*Disorder*) OR (depression) OR (dysthimia) OR (dysthimic disorder) OR ("depressive disorder") OR (anxiety) OR (anxiety*disorder*) OR ("Panic disorder") OR ("panic attack") OR ("separation anxiety") OR ("Acute distress") OR ("post traumatic stress disease") OR (PTSD) OR (post-traumatic stress disease) OR ("bipolar disorder") OR (bipolar*disorder*type*) OR (bipolar) OR ("manic-depressive") OR (manic episode) OR (depressive episode) OR (hypomanic episode) OR (schizophrenia) OR (schizoaffective) OR (schizophreniform)) AND (("substance use") OR ("substance abuse") OR ("drug abuse") OR ("substance-induced"))

SUPPLEMENTARY FILE 2: SUPPLEMENTARY TABLES

Table S1: Included studies looking at schizophrenia, substance abuse and neuroimaging changes.

	Author	DOI	Type of study	Year	MR Strength (T)	MR study type	Size of cohort(F/M)	Type of cohort	Group size	ROI	Findings	Substances used
1	Abush	10.1016/j.pscychresns.2018.03.008	Case control	2018	3	MRI	109(57.1/51.9)	(HC/SZ/BP) vs. cannabis use (ACU/NonACU)	HC-NonACU (n = 32) HC-ACU (n = 14) PSY-NonACU (n = 35) PSY-ACU (n = 28)	Whole brain, lt medial frontal gyrus, rt middle frontal gyrus, rt inferior frontal gyrus, rt inferior temporal gyrus, rt superior temporal gyrus, rt middle occipital gyrus, lt parahypocampal gyrus, rt parahypocampal gyrus, lt/rt lingual gyrus, rt precuneus, lt cuneus, rt cuneus	SZ< HC, BD	Cannabis
2	Alexander	10.1007/s00213-019-05298-w	cross sectional	2019	3	MRI + DWI/TBSS	96(34/62)	SZ VS SZ+ ST VS SIP	SZ(18) SZ+ST (39) SIP (39)	lt planum temporale, rt OPC	SZ > SZ+ ST	STIMULANTS
3	Bangalore	10.1016/j.schres.2007.11.029	cross sectional	2008	1.5	MRI	81(29/48)	FSZ w CN VS FSZ w/ CN VS HC	FSZ w CN (15) FSZ w/ CN (24) HC (42)	lt HCC, rt PCC	FSZ w CN ↓ GM	CANNABIS
4	Bernier	10.1016/j.schres.2016.04.022	Cross-sectional	2016	4	MRI H-MRS	74 (22/52)	SZ+Substance use vs HC with substance use	SZ+SU (27), HC+SU (24), HC (23)	Not Specified	SZ+SU= GPC+PC ↑<br>Glu+Gln: HC < HC+SU<br>Myol and of Cr+PCr: SZ+SU< HC	Hallucinogens
5	Bourque	10.1016/j.pscychresns.2013.05.012	Transversal	2013	3	fMRI	49 (NS)	SZ vs MDD vs HC	SZ (14), MDD(14), HC (21)	occipital and right frontal regions, right hippocampus, left precentral, right superior parietal and right middle temporal gyri.<br>occipital cortex, bilateral inferior temporal cortex and right middle temporal cortex<br>occipital cortex, right inferior temporal cortex and right	MDD > (SZ, HC)<br>SZ >(MDD, HC)<br>HC > (SZ, MDD)	Not Specified

										frontal regions, left putamen right precentral gyrus and hippocampus and caudate nucleus bilaterally		
6	Buchy	10.1016/j.pscychresns.2016.06.001	Case control	2016	3	MRI	723(302/421)	CHR vs HC	CHR(519) HC (204)	Thallamus, hippocampus, amygdala	No significant relationship	Alcohol
7	Cohen	10.1017/S146114571100068X	Case control	2012	1.5	MRI	55(9/46)	HC vs Cannabis using vs FES vs Cannabis using FES	Control subjects (n=19) Cannabis-using subjects (n=17) FES (n=13) Cannabis using FES (n=6)	Cerebellar gray matter	↑ cannabis use= ↓ volume	Cannabis, alcohol, tobacco
8	Cookey	10.1089/brain.2018.0611	Cross Sectional	2018	1.5	MRI, DWI	21(7/14)	ALL	ALL(21)	FULL BRAIN	Younger alcohol and cannabis= ↓ lPH, lAMYGD,lTALAM	ALCOHOL AND CANNABIS
9	Crocker	10.1016/j.schres.2014.05.004	CROSS SECTIONAL	2014	3	MRS	103(26/77)	FEP VS HC VS METH	FEP(29) HC(45) METH (29)	mPFC	Glu: METH < FEP = HC.	METHANFETAMINE
10	Cullen	10.1016/j.pscychresns.2011.08.010	Cross-sectional	2011	1.5 and 3	DTI	83(20/63)	Smoking vs non-smoking vs non-smoking HC	Smoking patients(28), Non-smoking patients(15) Non-smoking HC(40)	Cerebellum, Frontal lobe, Temporal lobe, Occipital lobe, Parietal lobe, Brain stem, Total cortical, Whole brain	SZ ↓< controls	Tobacco
11	Cunha	10.1016/j.schres.2013.04.009	CROSS SECTIONAL	2013	1.5	MRI	186(93/93)	FEP+CU VS FEP VS HC	FEP+CU(28) FEP(78) HC (80)	lt inf/mid frontal gyrus	FEP=FEP+CU<HC	CANNABIS
										rt paraHC gyrus	FEP=FEP+CU<HC	
										lt lateral ventricle	FEP>HC	
										rt Th	FEP>HC	

12	Dekker	10.1016/j.pscychresns.2009.06.003	Cross Sectional	2010	3	DTI	36(0/36)	EA-CANNABIS VS LO-CANNABIS VS NO CANNABIS VS HC	EA-CANNABIS (10), LO-CANNABIS(8), NO CANNABIS (8), HC (10)	Left posterior corpus callosum	CANNABIS NAIVE VS EO-CANNABIS : ↓ FA LVL, AND WM LVL	CANNABIS
										Right posterior corpus callosum		
13	Deshmukh	10.1016/j.schres.2005.04.025	Case control	2005	1.5	MRI	206(NS)	HC vs SZwA vs Alcohol vs SZwA+AAP vs SZwA+TAP vs AlcoholRS vs AlcoholLS	HC(51) vs SZwA(19) vs Alcohol(25) vs SZwA+AAP(29) vs SZwA+TAP(13) vs AlcoholRS(11) vs AlcoholLS(31)	Caudate, putamen, N.Accumbens	↓ SZ<comorbid<alcoholics	Alcohol
14	Domen	10.1017/S0033291718001320	Longitudinal	2019	3	MT-TD	Baseline: 258(126/132)	HC vs siblings vs Patients	HC(80) vs siblings(93) vs Patients(85)	White matter	↑Cannabis exposure= ↓ WM FA	Cannabis, alcohol
							Follow up: 159(71/ 88)	HC vs siblings vs Patients	HC(49) vs siblings(55) vs Patients(55)	right rostral and caudal anterior cingulate cortex	EOS+CUD ↓< HC, EOS	Cannabis
15	Ebdrup	10.1503/jpn.090049	Cross-sectional	2010	3	MRI VOXEL-WISE ANALYSIS	81(25/56)	SUD vs nSUD vs HC	SZ+SUD(9),SZ+ nSUD(29), HC(43)	lt/rt HCC	SZ+SUD↓ < HC	Not specified
										lt/rt caudate, lt/rt NACC	SZ+nSUD ↓ < HC	
										Left putamen, right putamen, intracranial, total brain, total gray matter, total white matter, left lateral ventricle, right lateral ventricle, third ventricle	Not significant	

16	Edith	10.1001/archpsyc.57.9.894	Transversal	2000	1.5	3D T1-weighted	132(0/132)	HC VS SZ VS COMORBID VS ALCOHOL	SZ(27) VS COMORBID(19) VS ALCOHOL(25) VS HC(61)	Fourth ventricle	↑ IN SZ THAN ALCOHOL	NS
										V1 gray matter	↓IN COMORBID THAN SZ	
										V1 white matter	↑ IN COMORBID THAN ALCOHOL	
17	Epstein	10.1016/j.schres.2014.04.035	Case control	2014	3	MRI	134(59/75)	HC vs CUD vs EOS vs EOS + CUD	HC (n = 53) CUD (n = 29) EOS (n = 34) EOS + CUD (n = 18)	right rostral and caudal anterior cingulate cortex	EOS+CUD ↓< HC, EOS	Cannabis
18	Epstein	10.1016/j.schres.2014.11.029	Case control	2015	3	MRI	79(36/46)	HC vs CUD vs EOSS	HC (n=29) CUD (n=19) EOSS (n=34)	Superior temporal lobe	CUD< EOS+CUD, HC	Cannabis
										Superior frontal gyri, inferior frontal regions, inferior parietal cortices, supramarginal gyri, inferior temporal gyri	EOS+CUD ↓< HC	
19	Epstein	10.1016/j.pscychresns.2014.10.010	cohort	2015	3	DTI	82(36/46)	EOSS VS CUD VS HC	EOSS(34) CUD(19) HC (29)	lt/rt Inferior longitudinal fasciculus, lt/rt inferior frontoccipital fasciculus, lt/rt corticospinal	EOSS<CUD<HC	CANNABIS
20	Fischer	10.1016/j.schres.2014.04.033	Case control	2014	3	f MRI	24(6/16, 2 NS)	HC vs cannabis vs THC	Healthy controls (N = 12) Cannabis (N = 6) THC (N = 6)	secondary visual cortex	SZ+CUD ↑ Connectivity	
										Ventral anterior cingulate cortex, orbitofrontal cortex, anterior cingulate cortex, anterior prefrontal cortex,	SZ+CUD ↓ connectivity	Cannabis

										orbitofrontal cortex, dorsolateral prefrontal cortex, subgenual cortex, parahippocampal cortex, entorrhinal, insular cortex, premotor cortex, primary motor cortex		
21	Gizewski	10.1111/j.1369-1600.2012.00437.x	cross sectional	2018	1.5	fMRI	57(NS)	SZ+ALC VS SZ VS ALC VS HC	SZ+ALC (12) SZ(21) ALC(12) HC (12)	vlPFC	↓ ACTIVATION IN SZ	ALCOHOL
										insular	↓ ACTIVATION IN SZ AND ALC	
22	Haller	10.1007/s10548-013-0288-8	Cross-sectional	2013	1.5	MRI DTI + VBM	50(14/36)	Cannabis-HD vs Cannabis-LD vs HC	Cannabis-HD(15), Cannabis-LD(18), HC(17)	Gray and white matter	No differences	Cannabis
23	Hartberg	10.1016/j.euroneuro.2017.11.019	Transversal	2018	1.5	MRI	591 (282/309)	CUD+ SZ/BD vs SZ/BD vs HC	CUD (132), No CUD (182), HC (277)	Right caudal middle frontal gyrus	CUD < NO CUD	Cannabis
										Right fusiform gyrus	CUD < NO CUD	
										Left superior gyrus	CUD < NO CUD	
24	James	10.1016/j.schres.2011.02.014	cross sectional	2011	1.5	VBM + Dti	60(20/40)	AOS + CU VS AOS VS HC	AOS + CU (16) AOS(16) HC (28)	Brain GM	↓ IN AOS	CANNABIS
										Longitudinal Fasciculi	↓ FA IN AOS+CU	
										Brain stem	↓ FA IN AOS+CU	
										Internal capsule	↓ FA IN AOS+CU	
										Corona radiata	↓ FA IN AOS+CU	
25	Jernigan	10.1001/archpsyc.1991.01810340013002	Transversal	1991	1.5	MRI	66 (19/47)	HC vs SZ	HC (24) vs SZ (42)	Infracallosal volume	SZ < HC	Cannabis
										Lenticular nucleus volume	HC < SZ	
										Mesial temporal and orbitofrontal regions	SZ < HC	
										Mesial temporal lobe gray matter	HC < SZ	

26	Joyal	10.1016/j.pscychresns.2003.09.003	Cross-sectional	2004	1.5	MRI	64(0/64)	SZ+SUD VS SZ VS HC	SZ+SUD(19), SZ(19), HC(26)	Intracranial	No differences	Alcohol
										Vermis, lt cerebellar	SZ<HC	
										rt cerebrellar, ant vermian, post vermian	SZ+CUD<SZ	
										Inferior vermian aerea	Not significant	
27	Joyal	10.1016/j.schres.2006.12.014	Case control	2007	1.5	f MRI	36(0/36)	HC vs SZ+PD+SUD vs SZ	HC(12) vs SZ+PD+SUD(12) vs SZ(12)	Brodman:9,10,11,44,45,46 and 47	SZ+APD+SUD↑>HC group	Alcohol
28	Jørgensen	10.1503/jpn.140163	Cross sectional	2015	1.5	MRI	743(349/394)	PSY+NIC VS PSY VS HC+NIC VS HC	PSY+NIC(250) PSY(256) HC+NIC (48) HC(189)	lt Ant rostrral ACC, lt ins	(Psy+NIC + HC+NIC) < PSY	NICOTINE
29	Koenders	10.1503/jpn.140081	Cross-sectional	2015	3	MRI	197(0/197)	CUD VS nCUD vs HC	CUD(80), nCUD(33), HC(84)	Total Cortical Grey Matter	No differences	Cannabis
										Hcc, Cd, ACC, parahippocampal, fusiform	NSD	
										Amygdala	CUD<nCUD<HC	
										Thalamus	CUD>nCUD+HC	
										Putamen	CUD+nCUD<HC	
										Orbito Frontal Cortex	nCUD<CUD	
										Insula	CUD+nCUD<HC	
30	Kumra	10.1016/j.jaac.2011.11.001	Cross-sectional	2012	3	MRI	115(54/61)	CUD VS SZ VS SZ+CUD VS HC	CUD(16), SZ(35), SZ+CUD(13), HC(51)	Left Superior Parietal Cortex	CUD>HC> SZ+CUD	Cannabis, tobacco, alcohol
										Left Medial Orbitofrontal Cortex lt med OFC, rt rostral midFC, lt rostral midFC, rt supFC, lt Th	CUD>HC> SZ+CUD	
										lt lat OFC, lt rostral ACC, lt HCC	NSD	
										Right Hippocampus	SZ+CUD> HC, CUD	

31	Lange	10.1017/S0033291716002920	Case control	2017	1.5	MRI	609(294/315)	SZ VS BD VS HC	SZ (n = 165) BD (n = 172) HC (n = 272)	Whole brain	↑ AUDIT-C score =↓ SZ and BD < HC	Alcohol
32	Liu	10.1007/s11682-017-9806-8	Cross sectional	2018	3	fMRI	63(15/48)	SZ-SM VS SZ VS HC-M	SZ-SM(21) SZ(21) HC-SM(21)	lt/rt Cd, mPFC, rt postcentral, rt supTC, lt precentral	nicotine = ↑ INTRINSIC BRAIN ACTIVITY	NICOTINE
33	Machielsen	10.1016/j.schres.2017.03.030	COHORT	2018	3	fMRI	58(/58)	SZ+CUD+RIS VS SZ+CUD+CLO VS SZ+RIS VS SZ+CLO VS HC	SZ+CUD+RIS(16) SZ+CUD+CLO(14) SZ+RIS(4) SZ+CLO(4) HC(20)	Ventral striatum	CUD= ↑ ACTIVATION, CLO= ↓ ACTIVATION, RIS= ↑ ACTIVATION	CANNABIS
										amygdala, insular, Th		
34	Malchow	10.1007/s00406-013-0451-y	Cross-sectional	2013	1.5	MRI + H1-MRS	77(34/45)	HC VS SZ	SZ(47), HC(30)	GM, WM, lt/rt HCC, lt amygdala, lt/rt Ncd	SZ<HC	Cannabis
										Rt Amydala, rt/lt putamen, lt/rt Th, All CC, CC seg. III, IV, V	NSD	
										Corpus Callosum Seg. I	SZ>HC	
										Corpus Callosum Seg. II	SZ+THC>SZ	
35	Mathalon	10.1001/archpsyc.60.3.245	Case control	2003	1.5	MRI	223(0/223)	SZ vs Alcohol vs comorbid vs HC	HC(62), Alcohol(62), SZ(64), comorbid (35)	prefrontal, frontal, anterior superior temporal	comorbid ↓< alcoholics, SZ only, and HC	
										posterior superior temporal, anterior parietal, and posterior parietal-occipital	NS	Alcohol
36	Moran	10.1093/schbul/sbx085	CROSS SECTIONAL	2018	3	fMRI	39(19/20)	SZ+SM VS HC-SM	SZ+SM(20) HC-SM(19)	Left midline frontal: Superior frontal gyrus (BA8/9/10), Right midline frontal, posterior: superior frontal gyrus (BA8), Right midline frontal, anterior: Superior frontal gyrus (BA9/10)	HC-SM > SZ-SM	NICOTINE

37	Moran	10.1093/schbul/sbs149	CROSS SECTIONAL	2013	3	fMRI	73(38/35)	SZ+SM VS HC-SM	SZ+SM(36) HC-SM(37)	dACC + related circuits	↓ rsFC in SZ vs HC.	NICOTINE
38	Moser	10.1001/jamapsychiatry.2017.4741	Longitudinal	2018	3	DWI,fMRI	172 (60/112)	HC vs SZ vs BD	HC(48), BD (37), SZ(92)	Not Specified	subcortical volume is realted with substance use and cannabis use	Cannabis and Alcohol
39	Nesvag	10.1016/j.schres.2006.11.008	Cross Sectional	2007	1.5	MRI	69(19/50)	PT	PT(69)	MGM, MWM<br>Frontal WM, temporal GM	Alcohol <non-AU<br>Alcohol <non-AU	ALCOHOL
40	Peeters	10.1016/j.euroneuro.2015.08.007	Case control	2015	3	f MRI	284(184/100)	Patients vs Siblings vs HC	Patients (63), Siblings (73), HC(59)	dorsolateral prefrontal cortex connectivity	Patients< Siblings, HC	Cannabis, alcohol, tobacco and others(not specified)
41	Peters	NA	CROSS SECTIONAL	2009	1.5	DTI	(0/35)	SZ	SZ(35)	Anterior internal capsule<br>Uncinate fasciculus<br>Frontal	FA ↑ IN CANNABIS BEFORE 17 YEARS	CANNABIS
42	Postma	10.1007/s00213-006-0307-5	Case control	2006	1.5	f MRI	56(0/56)	PS vs PnS vs HCS vs HCnS	PS, PnS, HCS, HCnS: NS	Not specified	PS↑> HC	Tobacco
43	Potvin	10.1080/15504263.2018.1526432	Case control	2019	3	f MRI	42(NS)	Smokers vs SSZ vs HC	Smokers(24), SSZ(18), HC(NS)	Bilateral nucleus accumebns	SSZ↑ >smokers, HC	Tobacco
44	Potvin	10.1016/j.schres.2016.03.011	Cross-sectional	2016	3	fMRI	42(29/13)	SZ+tobacco vs HC+tobacco	SZ+tobacco(18), HC+tobacco(24)	Rt caudate head, lt lingual gyrus<br>MedFC, rt angular gyrus<br>ACC, PCC, lt supFC, rt lingual gyrus	SZ>HC<br>NS<br>HC>SZ	Tobacco
45	Potvin	10.1016/j.pscychresns.2006.11.009	Cross-sectional	2007	1.5	fMRI	27 (8/19)	SZ + SUD vs HC	SZ + SUD (12), HC (15)	Ventral striatum grey matter density	SZ+SUD ↑	Not Specified

46	Quinn	10.1016/j.pscychresns.2018.08.002	Case control	2018	3	sMRI	246(74/172)	HC vs SZ+SUD vs SZ	HC(88), SZ+SUD(92), SZ(66)	Whole brain gray matter	SZ patients↓ < HC patients	Cannabis
47	Rais	10.1176/appi.ajp.2007.07071110	Cohort	2008	1.5	MRI	82(12/70)	SZNC VS SZC VS HC	SZNC(32) SZC(19) HC(31)	T5 cerebral GM, 3rd ventricle	SZC<SZNC or HC	
48	Rais	10.1016/j.euroneuro.2010.08.008	COHORT	2010	1.5	MRI	50(6/44)	SZ+CUD VS HC	SZ+CUD(19) HC(31)	rt SMA, rt infFC, rtOC, rt supTC, rt angular gyrus, rt PC, lt dlPFC, lt ACC, lt OC	SZ<HC	CANNABIS
										Whole cortex	Mean cortical thickness: SZ<HC	
49	Rapp	10.1016/j.pscychresns.2013.06.006	Cross-sectional	2013	1.5	MRI	60(39/21)	ARMS VS FEP	ARMS(37), FEP(23)	PCC	CU<non-CU	Cannabis, alcohol
										rt ACC Right Anterior Cingulum of Cannabis Users	cannabis users > non-CU	
50	Rigucci	10.1007/s00213-017-4745-z	CROSS SECTIONAL	2018	3	MRS	51(22/29)	EP+CUD VS HC	EP+CUD(18) HC (33)	mPFC	↓ Glu in EP+CUD	CANNABIS
51	Scheller-Gilkey	10.1016/s0920-9964(98)00096-6	Cross-sectional	1999	1.5	MRI	176(49/127)	SZ+SUD VS SZ	SZ(73), SZ+SUD(ALCOHOL: 11, others: 40, both:52)	NR	BD↑ MRI findings	Alcohol, THC, LSD, PCP, cocaine, BZD, mescaline, amphetamines
52	Schiffer	10.1093/brain/awq153	Case control	2010	1.5	MRI	51(0/51)	HC vs SUD vs SZ vs comorbid	HC(14), SUD(13), SZ(12), comorbid(12)	Gray matter	Comorbid ↓ < HC and SZ patients	Alcohol, cannabis

53	Smith	10.1002/hipo.22427	Case control	2015	1.5	MRI	97(43/54)	HC vs HC-CUD vs SZ vs SZ-SUD	HC (44), HC-CUD (10), SZ (28) SZ-CUD (15)	Hippocampus	SZ+CUD ↓	Cannabis
54	Smith	10.1016/j.schres.2011.05.014	Case control	2011	1.5	MRI	107(31/76)	HC vs SZ VS SZ-AUD	HC (n= 56) SZ (n= 35) SZ-AUD (n= 16)	Hippocampus, subcortical	HC↑>SCZ-AUD	Alcohol
55	Smith	10.1093/schbul/sbt176	Cross-sectional	2014	1.5	MRI	97(59/38)	CON VS CON+CUD VS SZ VS SZ+CUD	CON(44), CON+CUD(10), SZ(28), SZ+CUD(15)	Left Striatum	SZ+CUD<SZ	Cannabis, cocaine, hallucinogens, alcohol, stimulants, opioids, sedatives
										Right Striatum		
										Left Globus Pallidus		
										Right Globus Pallidus		
										Left Thalamus		
										Right Thalamus	CON+CUD <CON	
56	Solowij	10.1017/S003329171100050X	CROSS SECTIONAL	2011	3	MRI	39(0/39)	SZ+CUD VS CUD VS HC	SZ+CUD(8) CUD(15) HC(16)	Cerebellar WM	SZ AND CUD= ↓ WM THAN HC	CANNABIS
57	Solowij	10.1016/j.schres.2012.10.040	CROSS SECTIONAL	2013	3	MRI	39(0/39)	SZ+CUD VS CUD VS HC	SZ+CUD(8) CUD(15) HC(16)	left hippocampus	SZ+CUD ↑ areas of deflation	CANNABIS
										right hippocampus	SZ group ↓ VS HC	
58	Szeszko	10.1192/bjp.bp.106.024521	Cross-sectional	2007	1.5	MRI	107(29/78)	HC VS SZ+CUD VS SZ	HC(56), SZ+CUD(20), SZ(31)	sup FC GM, WM, ACC WM, OF WM	NS	Cannabis
										Total ACC Gm, Total OFC GM	SZ+CUD<SZ,HC	
59	Whitfield-Gabrieli	10.1016/j.schres.2017.07.029	Case control	2018	3	f MRI	24(6/16)	HC vs Cannabis vs THC	HC(n = 12) Cannabis (n = 6) THC (n = 6)	Posteior cingulate cortex, Bilateral inferior parietal lobe, and Medial prefrontal cortex	Cannabis alters the connectivity	Cannabis
60	Wobrock	10.1007/s00406-008-0831-x	Cohort	2009	1.5	MRI	41(14/27)	SUD vs nSUD(psychosis)	SUD(20),nSUD(21)	superior temporal gyrus/heschl's gyrus	Schizoaffective↑	
										Amygdala-hippocampal complex, , and cingulate gyrus	No relation	Cannabis, stimulants, opiates,

	Author	DOI	Type of study	Year	MR Strength(T)	MR study type	Size of cohort(F/M)	Type of cohort	Group size	ROI	Findings	Substances used
												hallucigens, alcohol
61	Wojtalik	10.3389/fpsyt.2014.00001	Cross-sectional	2014	3	fMRI	57 (29/38)	SZ vs SZ + SUD vs HC	SZ (20), SZ+SUD(17), HC (20)	L superior temporal gyrus	Activation = HC < SUD	Various drugs
										L superior parietal lobule	SZ+SUD < SZ	
										L cerebellum tonsil		
										L cerebellum pyramid		
										R supramarginal gyrus		
										Lt middle TC, lt angular gyrus, lt PCC	HC < SUD	
										lt PCC	General activation: SZ+SUD < SZ	
62	Zhang	10.1016/j.biopsych.2010.06.018	Cross-Sectional	2010	3	DTI	115(15/100)	SZ+Smoking vs SZ vs Smoking Controls vs HC	SZ+tobacco(32), SZ(14), Tobacco(48), HC(21)	Left Anterior Thalamic Radiation/Anterior Limb of Internal Capsule	Nonsmoking controls >SZ and smoking controls	Tobacco
										Uncinate Fasciculus/Inferior Fronto-Occipital Fasciculus		
										Left Frontal Cortex		

MRI: Magnetic resoannce imaging fMRI: Functional magnetic resonance imaging (H-)MRS: (Hydrogen-based) magnetic resonance spectroscopy NS: Not specified BD: Bipolar disorder, ARMS: At ristk mental state FEP: First episode psychosis SZ: Schizophrenia, EOS: Early onset schizophrenia, SUD: Substance use disorder, SIP: Substance-induced psychosis CUD: Cannabis use disorder ACC: Anterior cingualte cortex FHBD: Family history of bipolar disorder DEP: Depressive patients SZ: Schizophrenic patients AUD: Alcohol use disorder vlPFC: Ventrolateral prefrontal cortex GM: Gray matter Glu: Glutamate Gln: Glutamine Myo-Ins: Myoinositol Cho: Choline FC: Frontal cortex GABA: γ-aminobutyric acid HCC: Hipocampus dlPFC: dorsolateral prefrontal cortex oPFC: orbital prefrontal cortex NR: Not reported AUDIT: Alcohol use disorders identifiaction test CRAFFT: "Car Relax Alone Forget Friends Trouble" test

Table S2: Included studies looking at bipolar disorder, substance abuse and neuroimaging changes.

	Author	DOI	Type of study	Year	MR Strength(T)	MR study type	Size of cohort(F/M)	Type of cohort	Group size	ROI	Findings	Substances used
1	Altamura	10.1016/j.eurpsy.	Case control	2016	3	MRI	97(40/57)	BDns vs BDws vs SIP vs HC	BDns(17) vs BDws(10) vs SIP(16) vs	Whole brain gray matter	BDns and BDws<HC	Cannabis, cocaine, polyabusers

		2016.09.009							HC(27) vs HC PET (27)			
2	Bitter	10.1111/add.12668	Cross-sectional	2014	4	f MRI	67 (32/35)	HC vs CUD vs BD vs BD+CUD	HC (15), CUD (13), BD (14), BD+ CUD (25)	rt Amygdala, lt TH	BD > BD+CUD	NS
										Left nucleus accumbens	BD > BD+CUD, BD > HC, CUD > HC	
										Right thalamus	BD > BD+CUD, BD > HC	
										Left striatum	BD > HC, CUD > HC	
3	Bitter	10.1080/15504263.2013.869077	Cross-sectional	2014	4	MRS	67(32/35)	BD VS CU VS BD+CU VS HC	BD(14) CU(13) BD+CU (25) HC(15)	lt vlPFC	(BD and BD+CU)<(CU and HC)	Cannabis
4	Chitty	10.1016/j.jpsychires.2014.03.024	Case control	2014	3	H-MRS	172(119/53)	BD vs. HC	ACC BD(57), ACC HC(44), Hippocampus BD(38), Hippocampus HC(33)	ACC - GSH	NSD	Alcohol and tobacco
										HCC- GSH	↑AUDIT score= ↓GSH in BD patients	
5	Jarvis	10.1089/cap.2008.033	Cross-sectional	2008	3	MRI	14(9/5)	BD+CUD vs BD	BD+CUD(7), BD(7)	Precentral, right caudate gyrus volume, right middle occipital	BD+CUD>BD	Cannabis

										gyrus, right fusiform gyrus, cerebellar vermis		
										Left fusifrom gyrus volume	BD+CUD<BD	
6	Kirsch	10.1038/s41598-020-80407-w	Cross-sectional	2021	3	MRI	45(36/9)	FHBD VS FHBD+DEP VS HC	FHBD(9) FHBD+DEP(12) HC (26)	Prefrontal GM Paralimbic GM insular PFC dlPFC	↑ CTQ= ↓ ventral PFC GMV, left rostral PFC GMV, bilateral dlPFC and left insular GMV	Alcohol and cannabis
7	Lange	10.1017/S0033291716002920	Case control	2017	1.5	MRI	609(455/154)	SZ vs BD vs HC	SZ (165) BD (172) HC(272)	Whole brain	SZ and BD<HC ↑AUDIT-C score=↓cortex volume	Alcohol
8	Lippard	10.1002/jnr.23901	Longitudinal	2017	3	MRI	30(15/15)	BD with low CRAFT scores vs. BD with high CRAFT scores	low CRAFFT scores (11), high CRAFFT scores (19)	Gray matter	High CRAFFT scores < low CRAFFT scores	Alcohol and tobacco
9	Nery	10.1016/j.jpsychires.2009.09.006	Cross-sectional	2010	1.5	MRS	91(71/20)	BD-AUD VS HC	BD-AUD(23) HC(54)	lt dlPFC (Glu, Gln, Myo-Ins, Cho)	HC> BD-AUD	Stimulants and alcohol
10	Nery	10.1016/j.neulet.2011.08.026	Cross-sectional	2011	1.5	MRI	46(31/15)	BD-AUD VS HC	BD-AUD(21) HC(25)	lt medial FC, rt ACC	BD-AUD < HC	Alcohol

11	Prisciandro	10.1038/tp.2017.141	Transversal	2017	3	H-MRS	78(NS)	BD+AUD VS BD VS AUD VS HC	BD+AUD(20), AUD(20), BD(19), HC(19)	dACC (GABA)	BD+AUD<(AUD, BD, HC)	Alcohol
12	Shad	10.1016/j.comppsych.2014.04.016	Cross-sectional	2015	1.5	MRI	33(31/2)	BD+alcohol vs BD-alcohol	BD+alcohol(25), BD-alcohol(8)	Lt/rt HCC, lt/rt dlPFC, lt /rt oPFC	NSD	Alcohol
13	Strakowski	10.1016/0006-3223(93)90140-9	Cross-sectional	1993	1.5	MRI	33(18/15)	Patients vs HC	Patients(18), HC(15)	NR	NSD	NS

MRI: Magnetic resoannce imaging fMRI: Functional magnetic resonance imaging (H-)MRS: (Hydrogen-based) magnetic resonance spectroscopy NS: Not specified BD: Bipolar disorder BDns: Bipolar patients without substance abuse BDws: Bipolar patients with substance abuse SIP: Substance-induced psychosis CUD: Cannabis use disorder ACC: Anterior cingualte cortex FHBD: Family history of bipolar disorder DEP: Depressive patients SZ: Schizophrenic patients AUD: Alcohol use disorder vlPFC: Ventrolateral prefrontal cortex GM: Gray matter Glu: Glutamate Gln: Glutamine Myo-Ins: Myoinositol Cho: Choline FC: Frontal cortex GABA: γ-aminobutyric acid HCC: Hipocampus dlPFC: dorsolateral prefrontal cortex oPFC: orbital prefrontal cortex NR: Not reported AUDIT: Alcohol use disorders identifiaction test CRAFFT: "Car Relax Alone Forget Friends Trouble" test

<u>Table S3:</u> Included studies looking at depressive disorder, substance abuse and neuroimaging changes.

	Author	DOI	Type of study	Year	MR Strength(T)	MR study type	Size of cohort(F/M)	Type of cohort	Group size	ROI	Findings	Substances used
1	Chye	10.1016/j.pnpbp.2017.07.017	Cross-sectional	2017	NR	MRI	274(85/189)	HC vs CU	HC(128) CU (146)	OFC	Type 3 OFC pattern related to increased substance use and depressive symptoms	Alcohol, cannabis

2	Cornellius	10.1016/j.addbeh.2010.02.004	COHORT	2010	3	fMRI	6(1/5)	MDD+CU	All patients(6)	Amygdala	CU correlates with decreased activation	Cannabis
3	Kushnir	10.1017/S1461145710000696	Cross-sectional	2013	3	f MRI	18(6/12)	Depressive patients with tobbaco use (BOLD in smoking cues)	All patients (18)	Lt superior TC, Lt superior PC, lt Postcentral gyrus, rt Precentral gyrus, rt middle TC	Increased HAMD scores relates to increased activation	Tobacco
										Rt postcentral gyrus, Rt ins, Lt fusiform gyrus	NSD	
4	Li	10.1016/j.pscychresns.2017.09.001	CROSS SECTIONAL	2017	3	fMRI	56(NS)	KU vs HC	KU T (36) HC T (20)	subgenual ACC	CES-D scores correlated to increased connectivity	Ketamine
5	Martins	10.1016/j.drugalcdep.2018.08.006	Cross-sectional	2018	3	MRI	81 (N/A)	Lifetime vs current DCoP (DUD + MDD or PTSD)	DCoP (19), Non-DUD MDD (20), DUD (22), Trauma control (20)	right rostral ACC/ medial PFC	rACC/mPFC-TPJ connectivity: Lifetime = Current DCoP	Various drugs
										left temporal-parietal junction	NSD	
6	Osuch	10.1111/acps.12629	Cross-sectional	2016	3	rs-fMRI	74(40/34)	MDD VS HC	MDD(54), HC(20)	Lt/rt TC, Lt/rt OC, Lt/rt Fusiform cortex, rt Precuneus, rt Culmen, rt ACC, lt superior FC	MDD+early onset cannabis>Late onset	Cannabis
7	Nichols	10.1016/j.nicl.2021.102575	Case control	2021	3	f MRI	73(39/57)	HC vs MDD vs Cannabis vs	HC(20), MDD(20), CANNABIS(20),	ventrolateral prefrontal cortex, dorsolateral	MDD and cannabis use alter emotional processing	Cannabis

								MMD+cannabis	MDD+cannabis(17)	prefrontal cortex and dorsomedial prefrontal cortex		
8	Radoman	6538<:=4x6896 :256>255<5929	CROSS SECTIONAL	2019	3	MRI	118(58/60)	MDD+CUD VS CUD VS HC(48)	MDD+CUD(24) CUD(46) HC(48)	lt/rt mOFC, lt/rt superior OFC, lt/rt superior FC<br>Lt/rt midtmporal gyrus, rt entorhinal cortex	CUD<(MDD, HC)<br>(CUD + MDD)< (CUD, HC)	CANNABIS
9	Sjoerds	10.1017/S0033291713002274	Cross-sectional	2014	3	fMRI	65(30/35)	AD VS HC VS D/A	AD (31) HC(16), MDD+AUD(18)	Lt Putamen, rt/lt Th	MDD+AUD>(AD, HC)	ALCOHOL
10	Thayer	10.1016/j.pscychresns.2019.02.001	PILOT STUDY	2019	3	MRI	56(27/29)	Non-CUD vs. CUD	NU(28) CU (28)	lt lingual cortex, rt mFC<br>CSF, GM,WM, brainstem, lt/rt nACC, lt/rt amygdala, lt/rt NC, lt/rt HCC, lt/rt Pallidum, lt/rt putamen, lt/rt Th	CU>NU<br>NSD	CANNABIS
11	Viswanath	10.1016/j.neuropharm.2014.12.030	Case control	2015	3	MRI+DTI	151(66/85)	non-SUD vs SUD	NS	inferior, medial, and superior frontal gyri, insula, striatum, and anterior cingulate cortex	Connectivity: ↑ SUD; Size: ↓SUD	Alcohol,tobacco, cannabis, cocaine, amphetamine, inhalants, sedatives, opioids

12	Yi	10.1016/j.drugalcdep.2019.01.047	Cross-Sectional	2019	7	f MRI	16(0/33)	OUD + Depressive symptoms vs HC	Depressive symptoms (16), HC (17)	Superior FC, Th, SMA, rt Caudate, paracingulate gyrus, pFC, lt PCC, ACC, mFC, infFC	OUD + DS < HC	Opiates
13	Zorlu	10.1016/j.jpsychires.2016.09.009	CROSS SECTIONAL	2017	1.5	MRI	72(42/30)	MDD+Sm VS MDD VS HC	MDD+Sm(25) HC(22) MDD(25)	lt midTC, lt Postcentral, rt insula, lt/rt superior TC	MDD>MDD-SM AND HC	NICOTINE

MRI: Magnetic resonance imaging, fMRI: functional MRI, DTI: Diffussion-tensor imaging, CU: Cannabis users, DUD: Drug use disorders, DCoP: DUD with psychiatric comorbidities, MDD: Major depressive disorder, AUD: Alcohol use disorder, NU:Non-drug users, DU: Drug users, MDD+Sm: Depressive patients with smoking, OFC: Orbitofrontal cortex, TC: Temporal cortex, PC: Parietal cortex, ins: Insula, ACC: ANterior Central cingulate, PFC: Prefrontal cortex, OC: Occipital cortex, FC: Frontal cortex, Th: Thalamus, mFC: Medial frontal cortex, HCC: Hippocampus, infFC: inferior Frontal cortex

<u>Table S4:</u> Included studies looking at anxiety disorder, substance abuse and neuroimaging changes.

	Author	DOI	Type of study	Year	MR Strength(T)	MR study type	Size of cohort(F/M)	Type of cohort	Group size	ROI	Findings	Substances used
1	Karch	10.1016/j.jpsychires.2007.07.016	Cross-sectional	2008	1.5	fMRI	32(0/32)	SUD vs HC	SUD(16), HC(16), HA(8), LA(8)	lt / rt superior FC (BA 6), lt middle FC (BA 8), rt inferior FC/Insula (BA 45,13)	HC>SUD, HA>LA	Alcohol
										lt inferior FC/insula (BA 45/44)	SUD>HC	

										lt Precentral Gyrus (BA 6)	HC>SUD,LA>HA	
										lt / rt inferior PC (BA 40)	LA>HA	
2	Kim	http://dx.doi.org/10.1016/j.jad.2016.03.055	CROSS SECTIONAL	2016	3	MRI+DTI	69(42/27)	PD VS PD+AUD	PD(49) PD+AUD(20)	Total WM	WM volume: PD=PD+AUD	Alcohol
										CC, Internal capsule, corona radiata, superior PC, Lateral OC, PCC	PD+AUD>PD	
3	Oh	10.1016/j.addbeh.2020.106457	Cross-sectional	2020	3	MRI, fMRI	154 (79/75)	Low drug users vs Problem users	LU (77), PU (77)	OFC, dorsal striatum, habenula	PU > LU	Various drugs
4	Uhlmann	10.1007/s00406-018-0870-x	Cross-sectional	2019	3	MRI	75 (24/51)	AUD- vs AUD + Anxiety/depressive symptoms (AUD+)	AUD - (40), AUD+ (35)	lt/rt GM, lt/rt HC, lt NAcc	AUD+ > AUD-	Alcohol
5	Viswanath	10.1016/j.neuropharm.2014.12.030	Case control	2015	3	MRI+DTI	151(66/85)	non-SUD vs SUD	NS	FC, insula, striatum, ACC	Connectivity: ↑ SUD; Size: ↓SUD	Alcohol
6	Wong	10.1038/tp.2015.48	Cross-sectional	2015	3	DTI	58(0/58)	Heroine vs HC	Heroine(26), HC(32)	lt Forceps Major, lt Anterior Thalamic Radiation, lt Corticospinal Tract, lt Inferior Fronto-Occipital Fasciculus, lt Inferior Longitudinal Fasciculus, lt Superior Longitudinal Fasciculus, lt Temporal Division of Superior	Not significant	Heroine

										Longitudinal Fasciculus, lt Uncinate Fasciculus		

fMRI: functional magnetic resonance imaging. MRI: magnetic resonance disorder. DTI: diffusion tensor imaging, SUD: substance use disorder, HA: high anxiety trait, LA: low anxiety trait, LU: low-frequency users, PU: problem users, AUD: alcohol use disorder, HC: healthy control groups, FC: frontal cortex, GM: gray matter, HP: Hippocampus, NAcc: Nucleus accumbens, ACC: anterior cingulate cortex, PC: parietal cortex, BA: Brodmann's area, PD: Panic disorder, WM: White matter, CC: Cingulate cortex, OC: Occipital cortex

<u>Table S5:</u> Included studies looking at anxiety disorder, substance abuse and neuroimaging changes

.

	Author	DOI	Type of study	Year	MR Strength(T)	MR study type	Size of cohort(F/M)	Type of cohort	Group size	ROI	Findings	Substances used
1	De Bellis	10.1097/01.alc.0000179368.87886.76	CROSS SECTIONAL	2005	1.5	MRI	42(18/24)	AUD VS HC	AUD(14) HC (28)	Intracranial volume, cerbral CSF, PFC GM, Th, Pons, Cerebellum	NSD	Alcohol
										PFC volume	Adolescent-onset AUD < HC	
										PFC GM	AUD=HC	
										PFC WM	Adolescent-onset AUD < HC	
2	Harlé	10.1016/j.pscychresns.2020.111172	Case control	2020	3	f MRI	31(5/26)	SS vs PE	SS Group (n=17) PE Group (n=14)	Whole brain	↓ CAPS= ↑ deactivation	Alcohol

											in response to stimulus	
4	Schuff	10.1016/j.pscychresns.2007.04.011	Case control	2008	1.5	MRI + H MRSI	104(12/92)	P+A+ vs P+A− vs P−A+ vs P−A−	P+A+(28) P+A−(27) P−A+(23) P−A−(26)	HCC, ACC, frontal Wm	NSD	Alcohol

MRI: Magnetic resonance imaging. HMRS: Hydrogen-based magnetic resonance spectroscopy. fMRI: functional magnetic resonance imaging, AUD: Alcohol use disorder, HC: healthy control, HCC: Hippocampus, PE: prolonged exposure treatment plan, SS: "Seeking safety" behavior trait, CAPS: Clinician-administered PTSD scale score, P+: PTSD, A+: Alcohol use, P+A+: Comorbid PTSD and Alcohol used GM: Gray matter, WM: white matter, ACC: ANterior cingulate cortex, NSD: No significant differences,
